# Unsupervised gene set testing based on random matrix theory

**DOI:** 10.1186/s12859-016-1299-8

**Published:** 2016-11-04

**Authors:** H. Robert Frost, Christopher I. Amos

**Affiliations:** Department of Biomedical Data Science, Geisel School of Medicine, Dartmouth College, Hanover, 03755 NH USA

**Keywords:** Gene set testing, Pathway analysis, Random matrix theory, Tracy-Widom, Marc̆enko-Pastur

## Abstract

**Background:**

Gene set testing, or pathway analysis, is a bioinformatics technique that performs statistical testing on biologically meaningful sets of genomic variables. Although originally developed for supervised analyses, i.e., to test the association between gene sets and an outcome variable, gene set testing also has important unsupervised applications, e.g., *p*-value weighting. For unsupervised testing, however, few effective gene set testing methods are available with support especially poor for several biologically relevant use cases.

**Results:**

In this paper, we describe two new unsupervised gene set testing methods based on random matrix theory, the Marc̆enko-Pastur Distribution Test (MPDT) and the Tracy-Widom Test (TWT), that support both self-contained and competitive null hypotheses. For the self-contained case, we contrast our proposed tests with the classic multivariate test based on a modified likelihood ratio criterion. For the competitive case, we compare the new tests against a competitive version of the classic test and our recently developed Spectral Gene Set Enrichment (SGSE) method. Evaluation of the TWT and MPDT methods is based on both simulation studies and a weighted *p*-value analysis of two real gene expression data sets using gene sets drawn from MSigDB collections.

**Conclusions:**

The MPDT and TWT methods are novel and effective tools for unsupervised gene set analysis with superior statistical performance relative to existing techniques and the ability to generate biologically important results on real genomic data sets.

**Electronic supplementary material:**

The online version of this article (doi:10.1186/s12859-016-1299-8) contains supplementary material, which is available to authorized users.

## Background

Gene set testing, or pathway analysis, is a powerful and extensively employed approach for analyzing the output from large scale assaying techniques for nucleic acids and nucleic acid products, such as microarrays and high-throughput sequencing [1, 2]. By focusing the analysis on the association between a smaller number of functional gene sets and a specific clinical outcome, gene set testing can substantially improve statistical power, biological interpretation and replication relative to an analysis based on individual genomic variables [1, 3–5]. Given these advantages, researchers have invested significant effort in the last 10 to 15 years creating large gene set collections [6–8] and developing effective gene set testing methods [4, 9–12].

Gene set testing was originally developed for use in a supervised context, i.e., to quantify the association between a set of genomic variables, or genes, and a clinical outcome or phenotype. Typically, this is carried out via a two-stage process in which the association is first measured between each gene in the set and the phenotype, often using a simple linear regression model. The test statistics for all genes in the set are then combined into a gene set test statistic and significance is computed relative to the appropriate *H*_0_ and *H*_*A*_. Based on the form of *H*_0_ and *H*_*A*_, gene set testing methods are generally grouped into two main categories: self-contained tests and competitive tests [5, 13]. For self-contained tests, the null asserts that none of the gene set members have an association with the outcome, and for competitive tests, the null asserts that the members of the gene set are no more associated with the outcome than genes not in the set. In general, tests based on a competitive null hypothesis are viewed as more biologically relevant, and thus are far more commonly used, than those based on a self-contained null [5].

Although most applications of gene set testing involve a supervised model, a number of important unsupervised use cases exist, where unsupervised implies that testing is performed in the absence of an outcome variable. Important unsupervised gene set testing use cases include case-only analyzes and *p*-value weighting [14], which is explained in greater detail in the next paragraph. In prior work, we addressed the lack of effective unsupervised gene set testing techniques by developing the Spectral Gene Set Enrichment (SGSE) method [15] (see the SGSE paper for a detailed review of existing unsupervised approaches). The SGSE method computes unsupervised enrichment for each gene set via the association between gene set members and the principal components (PCs) of a genomic data set using the Principal Component Gene Set Enrichment (PCGSE) method [16] while taking into account the statistical significance of the eigenvalue associated with each PC according to a Tracy-Widom test [17].

One unsupervised use case of particular importance to the SGSE method, and the work detailed in this paper, is *p*-value weighting [14]. *P*-value weighting aims to reduce the burden on statistical power incurred by multiple hypothesis correction (MHC) by weighting the *p*-values computed for each hypothesis using weights that reflect the likelihood that the alternative hypothesis (*H*_*A*_) is true. As long as the weights are independent of the test statistics under the null hypothesis (*H*_0_), MHC methods will correctly maintain either the family-wise error rate (FWER) or false discovery rate (FDR). When *p*-value weighting is used with FDR methods, e.g., the Benjamini and Hochberg (BH) [18] method, the technique is referred to as weighted FDR control (wFDR). In the special case where the weights are binary, this approach is called screening-testing [19] and has the effect of selecting and testing just a subset of the original family of hypotheses.

Although widely applied for gene-environment interaction detection [20–23], *p*-value weighting can have a significant impact on gene set testing power given the significant growth in the size of common gene set collections, e.g., even the very selective Molecular Signatures Database (MSigDB) [8] now includes over 10,000 sets. For such large gene set collections, MHC can lower statistical power so substantially that it becomes impossible to identify true associations for many genomic data sets [24]. The link between *p*-value weighting and unsupervised gene set testing is based on the fact that an effective way to ensure the independence between data-driven weights and standard gene set test statistics under *H*_0_ is to ignore the outcome variable when computing the weight, i.e., base the weight on an unsupervised gene set test. In previously published research building on the SGSE method, we developed a screening-testing framework for gene set testing, called Spectral Gene Set Filtering (SGSF) [24], that computes binary weights using the *p*-values generated by the SGSE method.

Although it can be proven that weights based on an unsupervised gene set test (like the SGSE method) will be independent of common gene set test statistics under *H*_0_ (see proof in the Supplemental Material for [24]), this independence only ensures type I error control. To actually improve statistical power, the weight must also be associated with the test statistic under *H*_*A*_. For gene set testing, producing a data-driven weight therefore requires the use of an unsupervised test that can effectively identify the gene sets truly associated with the outcome of interest. As illustrated in the SGSF paper [24], statistics based on deviation from an identity population covariance structure represent a useful class of gene set weights that boost gene sets according to the empirical correlation among member genes. Although it is possible some biologically important gene sets exhibit little inter-gene correlation, it has been shown that groups of highly correlated genes are often associated with biological processes that play an important role in the measured experiment [11]. For example, the genes belonging to biological pathways have well characterized interactions and, if the pathway is active in a given experiment context, can be expected to exhibit correlated expression. This property is in fact used as the basis for computationally generating many gene sets, e.g., the MSigDB C4 cancer models [8, 25] were created via clustering of gene expression data.

While the output of the SGSE method is effective at identifying enriched gene sets for many use cases, there are several biologically important scenarios represented by non-identity covariance structures where the SGSE method will fail to select the gene sets truly associated with the output. For example, if different subgroups of a gene set are associated with different PCs or if all gene set members are associated with the same PC but the direction of association varies for different subgroups, the SGSE method, as well as unsupervised methods based on clustering of the genomic variables [26–28], will perform poorly. Such scenarios are biologically important and reflect many large gene sets associated with processes or pathways comprised by multiple distinct groups of genes where the action of the subgroups is either uncorrelated or counterbalancing (these use cases are represented by the multi-block and anti-correlated multi-block covariance structures, as detailed in Section “Simple covariance structure examples”). Another important limitation of the SGSE method is the fact that it only supports a competitive *H*_0_ and therefore cannot be used in cases where a self-contained test is of greater biological interest (see Sections “Self-contained gene set testing” and “Competitive gene set testing” below for detailed definitions of self-contained and competitive gene set tests).

To address the shortcomings of existing unsupervised tests and to support both self-contained and competitive tests across a wider range of biologically relevant data models, we have developed two novel unsupervised gene set tests, the Marc̆enko-Pastur Distribution Test (MPDT) and the Tracy-Widom Test (TWT) that are based on the covariance structure of the measured genomic variables. Although a number of existing gene set testing methods are also based on covariance structure analysis (e.g., GSCA by Choi and Kendziorski [29], GSNCA by Rahmatallah et al. [30], and GSA-SDR by Hsueh and Tsai [31]), with the exception of the SGSE method [15] and earlier cluster-based approaches [26–28], these methods are all supervised and identify interesting gene sets according to difference in the covariance structure between levels of a phenotype. The supervised nature of these tests means they cannot be used to support *p*-value weighting, case-only analyses or other unsupervised use cases.

Both the MPDT and TWT methods are based on random matrix theory (RMT) findings regarding the distribution of the eigenvalues of matrices with a *white* Wishart distribution [32, 33], i.e., the distribution of the sample covariance matrix for multivariate normal data with an identity population covariance matrix. As detailed in Section “Random matrix theory (RMT) benefits” below, these tests were based on RMT to provide better support for high-dimensional data, non-normal data and data based on small sample sizes. For MPDT, the test is based on the Marc̆enko-Pastur quarter-circle law characterizing the limiting empirical distribution of all of the eigenvalues of a *white* Wishart matrix. For TWT, the test is based on the Tracy-Widom law of order 1 characterizing the limiting distribution of the largest eigenvalue of a *white* Wishart matrix. Versions of the MPDT and the TWT are detailed in Section “Methods” for both self-contained and competitive null hypotheses. For the self-contained case, we contrast our proposed tests with the classic multivariate test based on a modified likelihood ratio criterion (MLRT) and, for the competitive case, we compare the new tests against a competitive version of the classic test and our SGSE method (also based on RMT).

As we demonstrate through simulation studies, the MPDT and TWT methods provide superior performance relative to the MLRT and SGSE methods on several biologically important covariance structures (e.g., the multi-block and anti-correlated multi-block structures detailed in Table 1). The practical utility of the TWT and MPDT methods is illustrated through a weighted FDR analysis of leukemia [34] and p53 [4] gene expression data sets relative to MSigDB gene set collections [8]. The remainder of this paper is organized as follows: Section “Methods” specifies the statistical properties of the MPDT and TWT methods, models for important use cases, simulation study design and approach for real data analysis, Section “Results” contains the results of the simulation studies and real data analyses and Section “Discussion” provides a discussion. Additional file 1 contains additional results for the real data analysis.

**Table 1:** Covariance structure examples

Name	Model	Self-contained	Competitive
Identity	$ \boldsymbol {\Sigma } =\left [\begin {array}{cc} I & 0 \\ 0 & I \\ \end {array}\right ]$	Accept *H*_0_	Accept *H*_0_
**Scaled identity**	$ \boldsymbol {\Sigma } = \left [\begin {array}{cc} \alpha I & 0 \\ 0 & \alpha I \\ \end {array}\right ]$	**Reject** ***H***_**0**_	**Accept** ***H***_**0**_
Single block	$ \boldsymbol {\Sigma } = \left [\begin {array}{cc} \rho & 0 \\ 0 & I \\ \end {array}\right ]$	Reject *H*_0_	Reject *H*_0_
Multi-block	$\boldsymbol {\Sigma } = \left [\begin {array}{cc} \left [\begin {array}{cc} \rho & 0 \\ 0 & \rho \\ \end {array}\right ] & 0 \\ 0 & I \\ \end {array}\right ]$	Reject *H*_0_	Reject *H*_0_
Anti-correlated multi-block	$ \boldsymbol {\Sigma } = \left [\begin {array}{cc} \left [\begin {array}{cc} \rho & -\rho \\ -\rho & \rho \\ \end {array}\right ] & 0 \\ 0 & I \\ \end {array}\right ] $	Reject *H*_0_	Reject *H*_0_
Inverted single block	$ \boldsymbol {\Sigma } = \left [\begin {array}{cc} I & 0 \\ 0 & \rho \\ \end {array}\right ] $	Accept *H*_0_	^∗^Accept *H*_0_
Repeated single block	$ \boldsymbol {\Sigma } = \left [\begin {array}{cc} \rho & 0 \\ 0 & \rho \\ \end {array}\right ] $	Reject *H*_0_	^∗^Reject *H*_0_
**Compound symmetry**	$ \boldsymbol {\Sigma } = \left [\begin {array}{cc} \rho & \rho \\ \rho & \rho \\ \end {array}\right ]$	**Reject** ***H***_**0**_	**Accept** ***H***_**0**_

Examples where the self-contained and competitive tests give different answers are in bold. For the inverted single block structure, a two-sided competitive null would be rejected whereas the one-sided competitive *H*_*A*_ would be accepted. For the repeated block structure, *H*_0_ will be rejected since a random sample of *g* genes from among all *p* genes will likely include some pairs with 0 covariance

## Methods

### Data assumptions

It is assumed that measurements have been made for *p* genomic variables under *n* independent experimental conditions, e.g., expression levels of *p* mRNA molecules within tissue samples from *n* subjects. This data will be modeled as a sample of *n* independent observations from a *p*-dimensional random vector **x** with mean ***μ*** and covariance ***Σ***. Although the unsupervised gene set tests developed in this paper are robust to deviations from normality (see Section “Random matrix theory (RMT) benefits” below for details), it will be assumed that **x** can be well approximated by a multivariate normal distribution after appropriate transformation, i.e., $\mathbf {x} \sim \mathcal {N}(\boldsymbol {\mu }, \boldsymbol {\Sigma })$. This data can be held in an *n*×*p* matrix **X** whose elements *x*_*i*.*j*_ represent the measured value of genomic variable *j* under condition *i*. Let **C** represent the mean-centered version of **X**.

It is assumed that the *p* genomic variables have been annotated to a collection of *f* biologically-based sets of genomic variables or gene sets, e.g., Gene Ontology (GO) terms [6]. These annotations can be held in a *f*×*p* binary annotation matrix **A** whose rows represent the *f* biologically-based sets and whose cells *a*_*i*,*j*_ hold indicator variables whose value depends on whether an annotation exists between gene set *i* and genomic variable *j*.

For a given gene set, *i*, the *p* variables can be partitioned into two sets, *p*_*g*_ and *p*_*c*_, according to the indicator variables in row *i* of matrix **A** with *p*_*g*_ containing all variables that are members of the gene set, *p*_*c*_ containing the complement of the gene set and *p*_∗_ representing the set of all *p* variables. Let the number of variables in subset *p*_*g*_ be represented by *g* and the number in *p*_*c*_ be represented by *c*=*p*−*g*. If the variables are reordered such that the variables in the gene set, i.e., *p*_*g*_, are listed before the variables not in the gene set, i.e., *p*_*c*_, the population, ***Σ***, and sample, **S**, covariance matrices can be partitioned as:

1 $$  \boldsymbol{\Sigma} = \left[\begin{array}{cc} \Sigma_{p_{g},p_{g}} & \Sigma_{p_{g},p_{c}} \\ \Sigma_{p_{c},p_{g}} & \Sigma_{p_{c},p_{c}} \\ \end{array}\right], \mathbf{S} = \frac{1}{n} \mathbf{C}^{T}\mathbf{C} = \left[\begin{array}{cc} S_{p_{g},p_{g}} & S_{p_{g},p_{c}} \\ S_{p_{c},p_{g}} & S_{p_{c},p_{c}} \\ \end{array}\right]  $$

This same partitioning can be applied to the population and sample correlation matrices.

### Self-contained gene set testing

For a self-contained gene set test, only the genomic variables that are members of the gene set may be used to compute the test statistic. Informally, the null hypothesis for an unsupervised and self-contained test asserts that none of the genomic variables in the gene set, i.e., *p*_*g*_, are enriched relative to the genomic data matrix **X** more than would be expected at random. Importantly, in an unsupervised context this measurement of gene set enrichment is defined with respect to the distribution of the random vector **x** and not with respect to the association between the members of **x** and some other covariate.

Given the partitioning of the population and sample covariance matrices specified in (1), one possible formulation of an unsupervised and self-contained gene set test measures enrichment as departure from a null distribution of $\mathcal {N}(\boldsymbol {\mu }_{g}, \mathbf {I})$ for the *p*_*g*_ variables in the gene set. This can be formally defined using the following null and alternative hypotheses:

2 $$ \begin{array}{l} H_{0}: \boldsymbol{\Sigma}_{p_{g},p_{g}} = \mathbf{I}, H_{A}: \boldsymbol{\Sigma}_{p_{g},p_{g}} \neq \mathbf{I} \end{array}  $$

Under this null hypothesis, $n\mathbf {S}_{p_{g},p_{g}}$ has a *white* Wishart distribution, $\mathcal {W}(n-1, \mathbf {I})$, i.e., a Wishart distribution where the population covariance matrix is equal to the identity matrix.

In Sections “Classic modified likelihood ratio test (MLRT)” – “Self-contained Marc̆enko-Pastur Distribution Test (MPDT)” below, three different tests of these hypotheses are described, each based on the null distribution of a test statistic **T**_self_ that is a function of **X** and *p*_*g*_. The test detailed in Section “Classic modified likelihood ratio test (MLRT)” is the classic test of ***Σ***=**I** from multivariate statistics. Sections “Self-contained Tracy-Widom Test (TWT)” and “Self-contained Marc̆enko-Pastur Distribution Test (MPDT)” describe self-contained versions of two new unsupervised gene set tests, the Marc̆enko-Pastur Distribution Test (MPDT) and the Tracy-Widom Test (TWT). Both the MPDT and the TWT are based on random matrix theory (RMT) findings regarding the distribution of the eigenvalues of matrices with a *white* Wishart distribution [32, 33].

It is important to note that, for practical applications, rejection of the null hypothesis (2) at a given *α* may be of little interest to researchers given the limited biological information provided by self-contained tests and the general sensitivity of such tests to small deviations from the null [5]. As a consequence, gene set testing is almost always performed against a competitive null hypothesis. The self-contained tests described in Sections “Classic modified likelihood ratio test (MLRT)” through “Self-contained Marc̆enko-Pastur Distribution Test (MPDT)” were therefore developed primarily to provide statistics for use in competitive tests, as detailed in Section “Competitive gene set testing” below.

#### Classic modified likelihood ratio test (MLRT)

The classic test of self-contained hypotheses (2) for multivariate normal data is based on a modified likelihood ratio criterion [35]. Specifically, this criterion leads to the following self-contained test statistic:

3 $$ \mathbf{T}_{\text{self}}(\mathbf{X}, p_{g}) = n(\text{trace}(\mathbf{S}_{p_{g},p_{g}}) - log|\mathbf{S}_{p_{g},p_{g}}| - g)  $$

Under the asymptotic regime in which *n*→*∞* and *g* is fixed, this statistic has a *χ* ^2^ distribution with *g*(*g*+1)/2 degrees of freedom.

#### Self-contained Tracy-Widom Test (TWT)

The TWT is based on the Tracy-Widom law of order 1 characterizing the limiting distribution of the largest eigenvalue of a *white* Wishart matrix. Since $n\mathbf {S}_{p_{g},p_{g}}$ has a *white* Wishart distribution, $\mathcal {W}(n-1, \mathbf {I})$ under the null hypothesis (2), the Tracy-Widom result can be used as the basis for a self-contained gene set test. Specifically, the limiting distribution of a centered and scaled version of the largest eigenvalue of $n\mathbf {S}_{p_{g},p_{g}}$, $\hat {\lambda }_{1}$, under *H*_0_ (2) is given by the Tracy-Widom law of order 1 [17]:

4 $$  {\lim}_{n,g \to \infty, n/g \to \eta \geq 1} Pr(\frac{\hat{\lambda}_{1} - \mu(g, n)}{\sigma(g, n)} < x) = \mathcal{F}_{1}(x)  $$

where the scaling and centering terms are given by $\mu (g, n) = (\sqrt {n-1} + \sqrt {g})^{2}$ and $\sigma (g, n) = (\sqrt {n-1} + \sqrt {g}) \left (1/(\sqrt {n-1}) + 1/(\sqrt {g}) \right)^{1/3}$.

The self-contained version of the TWT therefore defines **T**_self_ as the scaled and centered version of $\hat {\lambda }_{1}$, as specified in (4), and tests for deviation from the Tracy-Widom law of order 1 distribution expected under *H*_0_ (2).

5 $$  \mathbf{T}_{\text{self}}(\mathbf{X},p_{g}) = \frac{\hat{\lambda}_{1} - \mu(g, n)}{\sigma(g, n)}  $$

One disadvantage of the TWT is that it considers only the principal eigenvalue of $n\mathbf {S}_{p_{g},p_{g}}$ and will therefore ignore the significance of eigenvalues $\hat {\lambda }_{i}, i \geq 2$.

#### Self-contained Marc̆enko-Pastur Distribution Test (MPDT)

The MPDT is based on the Marc̆enko-Pastur quarter-circle law characterizing the limiting empirical distribution of all of the eigenvalues of a *white* Wishart matrix [32, 33]. If it is assumed that $n\mathbf {S}_{p_{g},p_{g}}$ has rank *g*, the empirical distribution function of the eigenvalues, $\hat {\lambda }_{i}, i=1,\ldots,g$, of $n\mathbf {S}_{p_{g},p_{g}}$ under *H*_0_ (2) is given by:

6 $$  F_{\hat{\lambda}_{i}}(x) = \frac{1}{g} \sum_{i=1}^{g} 1(\hat{\lambda}_{i} \leq x)  $$

and the Marc̆enko-Pastur quarter-circle law holds that ${\lim }_{n,g \to \infty, g/(n-1) \to \gamma \in (0, \infty)} F_{\hat {\lambda }_{i}}(x) = G(x)$ where the density of *G*(*x*) is given by $g(x) = 1/(2\pi \gamma x) \sqrt {(b_{+} - x)(x-b_{-}}, b_{\pm } = (1 \pm \sqrt {\gamma })^{2}$. The self-contained version of the MPDT leverages this result by comparing the empirical distribution function $F_{\hat {\lambda }_{i}}(x)$ (6) against the expected Marc̆enko-Pastur distribution for *γ*=*g*/(*n*−1) using a two-sided, one-sample Kolmogorov-Smirnov test. This is based on the null distribution of a statistic, **D**, defined as the maximum difference between the two distribution functions:

7 $$  \mathbf{T}_{\text{self}}(\mathbf{X},p_{g}) = \mathbf{D} = \sup_{x} |(F_{\hat{\lambda}_{i}}(x) - G(x))|  $$

This test has the benefit, relative to the TWT, of accounting for all of the eigenvalues of $n\mathbf {S}_{p_{g},p_{g}}$. A limitation of the MPDT is that the $\hat {\lambda }_{i}$ are dependent, which results in an incorrectly large degrees-of-freedom for the Kolmogorv-Smirnov test and an inflated type I error rate unless the dependence structure can be accurately modeled [36]. Fortunately, this inflated type I error rate is only an issue for a strictly self-contained test. When the **T**_self_ from the MPDT is used in a competitive test (as described in Section “Competitive gene set testing” below), the incorrect degrees of freedom no longer poses a problem. This test is therefore primarily useful as a means of ranking the *f* gene sets (according to the magnitude of **D** (7)).

### Competitive gene set testing

For competitive and unsupervised gene set tests, two primary forms of null hypothesis are possible. The first type of competitive null asserts that a given gene set is no more associated with **X** than are the other gene sets defined in the annotation matrix **A**. The second type of competitive null asserts that the members of a given gene set are no more associated with **X** than would be expected for a random set of genomic variables of the same size. Although both forms of null hypothesis are valid and address important biological questions, we focus solely on that later form of competitive test in this paper. Not only does this form of competitive null provide results for a given gene set that are invariant to the size and composition of **A** but it can easily be used as the basis for a comparative analysis of multiple gene sets, e.g., rank the gene sets defined in **A** according to the *p*-values from competitive testing.

For this paper, then, the null hypothesis for an unsupervised competitive gene set test informally asserts that the genomic variables in the gene set, i.e., *p*_*g*_, are no more enriched relative to the variance structure of the matrix **X** than would be expected for a set of *g* genomic variables drawn at random from among all *p* variables, i.e., $p_{g \in p_{*}}$ or *p*_*g*∗_. The one-sided alternative hypothesis of interest informally asserts that the genomic variables in *p*_*g*_ are more enriched relative to the variance structure of the matrix **X** than would be expected for a random set of variables *p*_*g*∗_. These competitive hypotheses can be formally defined in terms of the cumulative distribution functions of the eigenvalues of the population covariance matrices $\boldsymbol {\Sigma }_{p_{g},p_{g}}$ and $\boldsymbol {\Sigma }_{p_{g*},p_{g*}}$. Specifically, under the competitive *H*_0_ the eigenvalues of $\boldsymbol {\Sigma }_{p_{g},p_{g}}$ and $\boldsymbol {\Sigma }_{p_{g*},p_{g*}}$ have identical cumulative distributions and, under the corresponding *H*_*A*_, the cumulative distribution of the eigenvalues of $\boldsymbol {\Sigma }_{p_{g},p_{g}}$ is point-wise smaller than the cumulative distribution of the eigenvalues of $\boldsymbol {\Sigma }_{p_{g*},p_{g*}}$, i.e., distribution of the eigenvalues of $\boldsymbol {\Sigma }_{p_{g},p_{g}}$ is shifted towards larger values relative to the eigenvalue distribution for $\boldsymbol {\Sigma }_{p_{g*},p_{g*}}$. Mathematically, these competitive hypotheses can be stated as follows:

8 $$ \begin{aligned} H_{0}: \forall_{x} F_{\lambda_{i}(\boldsymbol{\Sigma}_{p_{g}, p_{g}})}(x) = F_{\lambda_{i}(\boldsymbol{\Sigma}_{p_{g*}, p_{g*}})}(x) \\ H_{A}: \forall_{x} F_{\lambda_{i}(\boldsymbol{\Sigma}_{p_{g}, p_{g}})}(x) \leq F_{\lambda_{i}(\boldsymbol{\Sigma}_{p_{g*}, p_{g*}})}(x) \end{aligned}  $$

where $F_{\lambda _{i}(\boldsymbol {\Sigma }_{p_{g}, p_{g}})}(x)$ and $F_{\lambda _{i}(\boldsymbol {\Sigma }_{p_{g*}, p_{g*}})}(x)$ represent the eigenvalue cumulative distribution functions, as defined by (6) above, of the population covariance matrices $\boldsymbol {\Sigma }_{p_{g}, p_{g}}$ and $\boldsymbol {\Sigma }_{p_{g*}, p_{g*}}$. It is important to note that *H*_0_ (8) does not assert that either $\boldsymbol {\Sigma }_{p_{g}, p_{g}} = \mathbf {I}$ or that $\boldsymbol {\Sigma }_{p_{g*},p_{g*}} = \mathbf {I}$. Instead, the *H*_0_ (8) asserts that $\boldsymbol {\Sigma }_{p_{g}, p_{g}}$ and $\boldsymbol {\Sigma }_{p_{g}*, p_{g}*}$ have equivalent eigenvalue distributions. In other words, the *H*_0_ (8) can hold even if both $\boldsymbol {\Sigma }_{p_{g}, p_{g}}$ and $\boldsymbol {\Sigma }_{p_{g}*, p_{g}*}$ deviate significantly from the identity matrix as long as the deviation is equivalent when characterized by the eigenvalues of the matrices. This competitive *H*_0_ is quite distinct from the corresponding self-contained *H*_0_ (2) that asserts an identity population covariance matrix.

To test competitive hypotheses (8), we define competitive versions of the MLRT, TWT and MPDT tests that use the self-contained statistic **T**_self_ computed for both *p*_*g*_ and *p*_*g*∗_, where **T**_self_ is defined by either (3), (5) or (7). For these competitive tests, statistical significance relative to the competitive hypotheses (8) is computed using the following permutation testing procedure for each gene set defined in **A**:

- Compute **T**_self_(**X**,*p*_*g*_) according to (3), (5) or (7).
- From among all $p \choose g$ possible combinations of *g* variables from the set of all *p* variables, select *B* random combinations.
- For each combination, *p*_*b*_,*b*=1,…,*B*, compute **T**_self_(**X**,*p*_*b*_) according to (3), (5) or (7).
- Use the permutation distribution of **T**_self_(**X**,*p*_*b*_) to compute the *p*-value for hypotheses (8):
  9 $$ \begin{aligned} &Pr(\mathbf{T}_{\text{self}}(\mathbf{X}, p_{g*}) > \mathbf{T}_{\text{self}}(\mathbf{X}, p_{g}) | H_{0})\\ &= \frac{\sum_{b=1}^{B} 1(\mathbf{T}_{\text{self}}(\mathbf{X}, p_{b}) > \mathbf{T}_{\text{self}}(\mathbf{X}, p_{g}))}{B} \end{aligned}  $$

For standard gene set testing, i.e., testing in a supervised context, the use of such a gene-level permutation distribution, i.e., a distribution generated by permuting the assignment of genes to gene sets, is problematic and can lead to an inflated type I error rate [11]. Specifically, because the permutation distribution breaks the correlation structure of the gene set, the permutation distribution of a supervised gene set test statistic (i.e., a statistic that reflects the association of set members with an outcome variable) will have a much smaller variance than the true null distribution of the gene set test statistic given the correlation present among gene set members. As an example, suppose the supervised gene set statistic is the mean of the t statistics capturing association between genomic variables in the set and a binary outcome. If the members of a gene set are correlated, then the true null distribution of this gene set statistic has expectation 0 but a variance that is much larger than the variance of the statistic computed under the permutation null distribution. In an unsupervised context, however, breaking the correlation between gene set members through the permutation test does not pose a problem since the analysis is focused on the variance structure of the gene set members and not on their association with an outcome variable. In this case, the competitive null asserts a correlation structure among gene set members that matches what is generated through permutation, i.e., under *H*_0_ (8) the correlation structure for a gene set matches the correlation structure for a random sample of the same size.

### Random matrix theory (RMT) benefits

The development of the RMT-based MPDT and TWT methods was motivated by three key factors (which we validate and quantify through the simulation studies described in Section “Evaluation design”):

1. Asymptotic regime: The classic test holds under the standard asymptotic regime in which *n*→*∞* while *p* is fixed. The RMT-based tests, on the other hand, hold under an asymptotic regime where both *n* and *p*→*∞* while the ratio *p*/*n*→*γ*∈(0,*∞*). Importantly, RMT asymptotics can be expected to provide a more accurate approximation of genomic data than standard asymptotics [37].
2. Deviation from normality: Although both the classic test and the RMT-based tests are derived under the assumption of multivariate normality for **x**, the RMT-based tests, due to the universality properties of the RMT distributions, can be expected to be more robust to deviations from normality for the elements of **x** [38]. Such distributional robustness is especially important in the context of gene set testing of genetic variation data, e.g., genome-wide association data capturing single nucleotide polymorphisms (SNPs).
3. Small sample size: Although both the classic test and the RMT-based tests are asymptotic approximations, the RMT-based tests can be expected to perform better for very small samples sizes, e.g., Johnstone found that the distribution of the largest eigenvalue of a *white* Wishart matrix was well approximated by the Tracy-Widom law of order 1 for data sets as small as *p*=5 and *n*=20 [17].

### Simple covariance structure examples

The behavior of the self-contained and competitive tests outlined in Sections “Self-contained gene set testing” and “Competitive gene set testing” above can be illustrated using simple, but biologically meaningful, use cases based on different population covariance matrix structures. These covariance structures, along with expected results for the self-contained and competitive tests, are shown in Table 1 using the partitioning from Section “Data assumptions”. For simplicity, it is assumed that there is only one gene set containing the first *p*/2 genomic variables. Examples where the two tests give different answers are in bold. These covariance structures are referenced in Sections “Simulation design to assess type I error control”, “Simulation design to assess statistical power” and “Discussion” to characterize the simulation designs and discuss the relative performance of the evaluated methods.

### Evaluation design

#### Benchmark unsupervised and competitive gene set tests

For comparative evaluation of the competitive versions of the TWT and MPDT methods outlined in Sections “Self-contained gene set testing” and “Competitive gene set testing”, two benchmark unsupervised gene set tests were used: the classic modified likelihood ratio test (MLRT) and the Spectral Gene Set Enrichment (SGSE) [15] method. For the MLRT test, competitive testing used the classic modified likelihood ratio test statistic detailed in Section “Classic modified likelihood ratio test (MLRT)Classic modified likelihood ratiotest (MLRT)” with the competitive permutation procedure outlined in Section “Competitive gene set testing”. For all simulation and real data analyses detailed in this paper, the SGSE method was executed using all PCs with non-zero eigenvalues, gene-level test statistics set to the Fisher-transformed Pearson correlation coefficients between each gene and each PC, statistical association between each PC and each gene set computed using a correlation-adjusted two-sample t-test between the gene-level test statistics for gene set members and non-gene set members and overall unsupervised gene set association computed using the weighted Z-method on the PC-level *p*-values with weights set to the PC variance multiplied by the lower-tailed *p*-value from a Tracy-Widom test on that PC eigenvalue. See the original SGSE paper [15] for more information on the operation and configuration of the method.

#### Simulation design to assess type I error control

To assess type I error control for the competitive versions of the MPDT and TWT methods, as detailed in Section “Competitive gene set testing”, and the two benchmark competitive methods listed in Section “Benchmark unsupervised and competitive gene set testsBenchmarkunsupervised and competitive gene set tests”, data were simulated according to eight null simulation designs as outlined in Table 2 (seven for multivariate normal data and one for multivariate binomial data). In Table 2, the covariance structure column refers to one of the models listed in Table 1, *p* represents the total number of simulated genes, *n* represents the number of independent samples in each data set, *g* represents the size of each disjoint gene set, *σ* ^2^ represents the variance for all variables and *ρ* represents the pairwise covariance between all genes (i.e., all gene pairs have the same covariance irrespective of gene set membership). The multivariate binomial distribution was included to mimic single nucleotide polymorphism (SNP) data specified using additive coding. For all designs, 1,000 data sets were simulated and tested for unsupervised enrichment against a gene set annotation matrix **A** that defined *p*/*g* disjoint gene sets each containing *g* genes. For the competitive versions of the MLRT, MPDT and TWT methods, the number of permutations *B* was set to 500. For SGSE, all *g* principal components were used and other method parameters were specified as detailed in Section “Benchmark unsupervised and competitive gene set testsBenchmark unsupervised and comp-etitive gene set tests”.

**Table 2:** Simulation designs for type I error

Type I error design #	Covariance structure	*g*	p	n	*σ* ^2^	*ρ*
MVN-1	Identity	10	100	100	1	0
MVN-2	Scaled Identity	10	100	100	2	0
MVN-3	Compound symmetry	10	100	20	1	0.1
MVN-4	Compound symmetry	10	100	50	1	0.1
MVN-5	Compound symmetry	10	100	100	1	0.1
MVN-6	Compound symmetry	10	100	100	2	0.1
MVN-7	Compound symmetry	10	100	100	1	0.2
Binomial-1	Compound symmetry	10	100	100	0.375	0.1

Simulation design models for assessing type I error control using a multivariate normal distribution (MVN-1 thru MVN-7) or a multivariate binomial (*n*=2, *p*=0.25) distribution (Bionomial-1) for x

#### Simulation design to assess statistical power

To assess statistical power for the four evaluated methods, data was simulated according to ten different simulation designs outlined in Table 3 (nine designs for multivariate normal data and one for multivariate binomial data). The motivation and implications of each simulation model are discussed in more detail in Sections “Unsupervised gene set testing for the single block modelUnsupervised geneset testing for the single block model” – “Unsupervised gene set testing for non-normal dataUnsupervisedgene set testing for the single block model” below. With the exception of the *σ* ^2^ and *ρ* columns, the columns in Table 3 have the same interpretation as the corresponding columns in Table 2, as detailed in Section “Simulation design to assess type I error controlSimulationdesign to assess type I error control” above. For Table 3, *σ* ^2^ represents the variance of members of the non-null gene sets (a variance of 1 was used for all genes in null sets) and *ρ* represents the pairwise covariance between the members of non-null gene sets. All models listed in Table 3 included just a single non-null gene set with the exception of model MVN-9, for which all 10 disjoint gene sets were non-null. For model MVN-7, the non-null gene set was divided into 5 disjoint sub-blocks of size 2 with a covariance of 0.2 between the two members of each sub-block and 0 covariance between members of different sub-blocks. For model MVN-8, the non-null gene set was divided into two 5 member sub-blocks with pairwise covariance between sub-block members of 0.1 and pairwise covariance between members of different sub-blocks set to -0.1. Similar to the simulation procedure for assessing type I error control outlined in Section “Simulation design to assess type I error control”, 1,000 data sets were simulated for each of the ten designs and tested for unsupervised enrichment against a gene set annotation matrix **A** that defined one truly enriched gene set containing the first 10 variables. Configuration of the MLRT, MPDT, TWT and SGSE methods mirrored the settings specified for the type I error control simulations.

**Table 3:** Simulation designs for statistical power

Power design #	Covariance structure	*g*	p	n	*σ* ^2^	*ρ*
MVN-1	Single block	10	100	100	1	0.1
MVN-2	Single block	10	100	50	1	0.1
MVN-3	Single block	10	100	20	1	0.1
MVN-4	Single block	10	100	100	1	0.15
MVN-5	Single block	10	100	100	1.1	0
MVN-6	Single block	10	100	100	1.15	0
MVN-7	Multi-block	10	100	100	1	0.2/0
MVN-8	Anti-cor. multi-block	10	100	100	1	0.1/-0.1
MVN-9	Repeated block	10	100	100	1	0.1
Binomial-1	Single block	10	100	100	0.375	0.1

Simulation designs for assessing statistical power using a multivariate normal distribution (MVN-1 thru MVN-9) or multivariate binomial (*n*=2, *p*=0.25) distribution (Binomial-1) for x

#### Real data analysis design

To demonstrate the practical utility of the proposed methods, gene set testing was performed using a weighted FDR approach [14] on two classic gene expression data sets relative to v5.0 of the MSigDB gene set collections [8]. Specifically, the following MSigDB collections were tested: c1.all (positional), c2.cpg (curated: chemical and genetic perturbations), c2.cp (curated: canonical pathways), c3.mir (motif: microRNA targets), c3.tft (motif: transcription factor targets), c4.cgn (computational: cancer gene neighborhoods), c4.cm (computational: cancer modules), c5.bp (GO: biological process), c5.cc (GO: cellular component), c5.mf (GO: molecular function), c6.all (oncogenic signatures), c7.all (immunologic signatures). Prior to analysis, each MSigDB collection was filtered to remove gene sets with less than 5 or more than 200 members.

For the evaluations detailed in this paper, the proposed unsupervised gene set testing methods (TWT and MPDT) and the benchmark methods (SGSE and MLRT) were used to generate weights that were applied to the *p*-values generated via supervised gene set testing using the CAMERA method [11]. The weighted CAMERA *p*-values were then subjected to a wFDR analysis using the Benjamini and Hochberg (BH) [18] method. See Section “Configuration of CAMERA method for real data analysis” below for further details on the CAMERA method and the configuration settings used for these analyses. As detailed in Genovese et al. [14], the BH method provides valid FDR control when applied to a set of weighted *p*-values as long as the weights have an average value of 1 and are independent of the *p*-values under *H*_0_. To meet these requirements, the weights were based on the -log of the *p*-values generated by the evaluated competitive unsupervised gene set testing methods. If *u*_*i*_ represents the *p*-value from the unsupervised test for gene set *i* (out of a total of *f* gene set tests), the weights, *w*_*i*_, were calculated as $w_{i} = -log(u_{i})/(1/f \sum _{j=1}^{f} -log(u_{j}))$, which ensures that $\sum _{i=1}^{f} w_{i} = f$. If *s*_*i*_ represents the *p*-value from the CAMERA supervised test for gene set *i*, weighted *p*-values were then computed as $s^{*}_{i} = w_{i} s_{i}$ with the wFDR *q*-values computed using the standard BH method applied to $s^{*}_{i}$.

For TWT, MPDT and MLRT, the number of permutations *B* for competitive testing was set to 5000. For SGSE, all PCs associated with non-zero eigenvalues were used and other method parameters were specified as detailed in Section “Benchmark unsupervised and competitive gene set testsBenchmark unsupervised and competitive geneset tests”. The two classic gene expression data sets analyzed were the Armstrong et al. [34] leukemia gene expression data set and the p53 gene expression data set used in the 2005 GSEA paper [4]. These data sets were selected because of their easy accessibility and extensive use in the gene set testing literature [4, 9], factors that will allow other researchers to more easily replicate and interpret the results outlined in this paper. Similarly, v5.0 of the MSigDB collections were chosen for analysis due to their accessibility and high quality annotations.

#### Configuration of CAMERA method for real data analysis

CAMERA [11] is a two-stage, competitive gene set testing method that adjusts for the correlation between gene set members. CAMERA performs gene set testing using the following approach:

1. Model the relationship between the genomic variables *x*_*i*_,*i*=1,…,*p* and phenotype *y* using a series of *p* univariate linear models of the form *x*_*i*_∼*β*_0_+*β*_1_ **y**+***ε***. If multiple phenotype variables exist, a contrast of model coefficients must also be specified.
2. Compute gene-level test statistics, *z*_*i*_,*i*=1,…,*p*, from each of the *p* univariate models. The t-statistic associated with $\hat {\beta _{1}}$ is a typical choice. CAMERA uses a normalized t-statistic.
3. Use the gene-level test statistics to generate gene set test statistics, *S*_*j*_, for each of the gene sets in the target collection. The mean difference test statistic, which follows a t-distribution under *H*_0_, is a common choice: $S_{j} = (\bar {z}_{j} - \bar {z}_{j^{c}})/(\sigma _{p} \sqrt {\frac {1}{m_{j}} - \frac {1}{p-m_{j}}})$, where *m*_*j*_ is the number of genomic variables in set *j*, $\bar {z}_{j}$ is the mean of the *z*_*i*_ for members of gene set *j*, $\bar {z}_{j^{c}}$ is the mean of the *z*_*i*_ for genes not in set *j* and *σ*_*p*_ is the pooled standard deviation of the *z*_*i*_. CAMERA uses a correlation-adjusted version of the mean difference statistic.
4. Determine the statistical significance of the gene-level test statistics under null hypothesis that the *z*_*i*_ for genomic variables in the gene set are identically distributed to the *z*_*i*_ for genomic variables not in the gene set. CAMERA determines statistical significance using a two-sample t-test on the correlation-adjusted mean difference statistic. Many other two-stage competitive gene set testing methods use permutation of **y** to calculate a *p*-value.

For enrichment of the MSigDB gene set collections relative to the leukemia and p53 data, CAMERA was executed with default settings and gene-wise test statistics (*z*_*i*_ above) calculated via the linear regression of the gene expression value on a data set specific phenotype. For the leukemia data, the phenotype was the acute myeloid leukemia (AML) versus acute lymphoblastic leukemia (ALL) status while for the p53 data the phenotype was the p53 mutated status. For both data sets, false discovery rate (FDR) values were computed using the BH method [18] for both unweighted and weighted *p*-values.

## Results

### Simulation results

#### Type I error control results

Results from the type I error simulation studies detailed in Section “Simulation design to assess type I error control” are shown in Table 4. As listed in Table 4, type I error control was excellent for all evaluated methods on all eight null simulation designs.

**Table 4:** Average type I error rate

#	Cov. struct., n, *σ* ^2^, *ρ*	TWT	MPDT	MLRT	SGSE
MVN-1	Identity, 100, 1, 0	0.049	0.049	0.050	0.050
MVN-2	Scaled Identity, 100, 2, 0	0.050	0.045	0.049	0.052
MVN-3	Compound sym., 20, 1, 0.1	0.053	0.051	0.051	0.049
MVN-4	Compound sym., 50, 1, 0.1	0.049	0.045	0.048	0.051
MVN-5	Compound sym., 100, 1, 0.1	0.046	0.051	0.051	0.051
MVN-6	Compound sym., 100, 2, 0.1	0.048	0.051	0.048	0.046
MVN-7	Compound sym., 100, 1, 0.2	0.049	0.049	0.052	0.050
Binom-1	Compound sym., 100, 1, 0.1	0.044	0.054	0.053	0.048

Average type I error rate for each of the evaluated competitive methods computed on 1000 simulated data sets for the eight simulation designs detailed in Table 2

#### Power results

Results from the power simulation studies detailed in Section “Simulation design to assess statistical power” are shown in Table 5. Although no single competitive method was superior for all models, the TWT method had the best overall performance with the largest average power for six of the ten simulations and close to the best power for the remaining four models. While the TWT method was the most powerful in the majority of the use cases, each of the tested methods had the best power for at least one of the tested models. In all cases, the average relative power of the four methods was consistent with the expected behavior of the methods for the simulated covariance structures. Sections “Unsupervised gene set testing for the single block model” thru “Unsupervised gene set testing for non-normal data” below contain more detailed discussions of the results for each of these ten models.

**Table 5:** Average empirical power

#	Cov. struct, n, *σ* ^2^, *ρ*	TWT	MPDT	MLRT	SGSE
MVN-1	Single block, 100, 1, 0.1	**0.87**	0.35	0.79	0.76
MVN-2	Single block, 50, 1, 0.1	0.52	0.20	0.42	**0.55**
MVN-3	Single block, 20, 1, 0.1	0.24	0.16	0.18	**0.37**
MVN-4	Single block, 100, 1, 0.15	**0.99**	0.66	0.97	0.95
MVN-5	Single block, 100, 1.1, 0	**0.41**	0.38	0.16	0.10
MVN-6	Single block, 100, 1.15, 0	0.63	**0.68**	0.29	0.10
MVN-7	Multi-block, 100, 1, 0.2/0	0.31	0.28	**0.56**	0.19
MVN-8	Anti-cor. multi-block, 100, 1, 0.1/-0.1	**0.85**	0.32	0.74	0.06
MVN-9	Repeated block, 100, 1, 0.1	**0.49**	0.11	0.23	0.10
Binom-1	Single block,100, 1, 0.1	**0.64**	0.02	0.26	0.55

Average empirical power for each of the evaluated competitive methods computed on 1000 simulated data sets for the ten simulation designs detailed in Table 3. The largest average power found for each design is listed in bold

### Real data results

As outlined in Section “Real data analysis design”, the proposed and benchmark methods were evaluated via a wFDR analysis of leukemia [34] and p53 [4] gene expression data sets relative to 12 of the MSigDB v5.0 collections. For these analyses, the results of the unsupervised test were used to weight the *p*-values generated by supervised gene set testing. Table 6 contains the results for the C7 collection (immunologic signatures) relative to the leukemia data and Table 7 contains the results for the C6 collection (oncogenic signatures) relative to the p53 data. The first column in each table contains the gene set name with the number of genes in the set in parentheses. The second column lists the direction of enrichment, the third column ("GSE *p*-value) contains the enrichment significance as computed via the supervised CAMERA method and the forth column (“Unfiltered *q*-value”) holds the false discovery rate *q*-value based on the unweighted supervised GSE *p*-values. Columns five through eight display the *q*-values from a wFDR analysis using each of the evaluated unsupervised gene set testing methods to compute the *p*-value weight.

**Table 6:** Leukemia gene expression results

Gene set	Direction	GSE	Unweighted	MLRT	SGSE	TWT	MPDT
		*p*-value	*q*-value	wFDR	wFDR	wFDR	wFDR
*GSE10325_{B}CELL_{V}S_{M}YELOID_{U}P (124)	ALL	0.00225	0.999	0.655	0.711	**0.0969**	1
*GSE29618_{B}CELL_{V}S_{M}ONOCYTE_{D}AY … (130)	ALL	0.00302	0.999	0.655	0.711	**0.0969**	1
GSE29618_{B}CELL_{V}S_{M}DC_{D}AY7_{F}LU … (126)	ALL	0.0046	0.999	0.655	0.729	0.574	1
GSE10325_{C}D4_{T}CELL_{V}S_{B}CELL_{D}N (132)	ALL	0.005	0.999	1	0.711	0.574	1
*GSE10325_{L}UPUS_{B}CELL_{V}S_{L}UPUS_ … (123)	ALL	0.00563	0.999	0.431	0.711	**0.12**	1
GSE29618_{B}CELL_{V}S_{M}DC_{U}P (133)	ALL	0.00719	0.999	0.655	0.955	0.574	1
GSE29618_{B}CELL_{V}S_{M}ONOCYTE_{U}P (108)	ALL	0.00776	0.999	0.655	0.711	0.574	1
GSE29618_{B}CELL_{V}S_{M}ONOCYTE_{D}AY … (143)	AML	0.0137	0.999	0.655	0.711	0.574	1
GSE24634_{T}REG_{V}S_{T}CONV_{P}OST_{D}A … (123)	AML	0.0162	0.999	1	0.711	1	1
GSE6269_{H}EALTHY_{V}S_{S}TREP_{A}UREU … (133)	AML	0.0168	0.999	0.655	0.711	0.574	1
GSE29618_{B}CELL_{V}S_{M}DC_{D}AY7_{F}LU … (126)	AML	0.0171	0.999	0.655	0.711	1	1
GSE6269_{H}EALTHY_{V}S_{S}TREP_{P}NEUM … (134)	AML	0.0174	0.999	0.655	0.711	0.742	1
GSE15767_{M}ED_{V}S_{S}CS_{M}AC_{L}N_{U}P (117)	AML	0.0229	0.999	1	0.711	1	1
GSE6269_{E}_COLI_{V}S_{S}TREP_{A}UREUS … (130)	AML	0.0245	0.999	1	0.711	1	1
GSE22886_{N}AIVE_{C}D8_{T}CELL_{V}S_{N}E … (122)	AML	0.0295	0.999	1	0.729	1	1
GSE6269_{F}LU_{V}S_{E}_COLI_{I}NF_{P}BMC … (128)	AML	0.0306	0.999	1	0.711	1	1
GSE29618_{M}ONOCYTE_{V}S_{P}DC_{U}P (126)	AML	0.0333	0.999	0.659	0.711	0.706	1
GSE6269_{H}EALTHY_{V}S_{S}TREP_{A}UREU … (109)	ALL	0.0353	0.999	0.906	0.907	0.592	1
GSE3982_{M}EMORY_{C}D4_{T}CELL_{V}S_{B}C … (73)	ALL	0.0361	0.999	1	0.995	1	1
GSE360_{C}TRL_{V}S_{M}_TUBERCULOSIS_ … (71)	AML	0.0364	0.999	1	0.711	1	1
GSE11057_{E}FF_{M}EM_{V}S_{C}ENT_{M}EM_{C} … (85)	AML	0.0381	0.999	1	0.729	1	1
GSE10325_{L}UPUS_{B}CELL_{V}S_{L}UPUS_ … (88)	AML	0.0384	0.999	0.655	0.711	0.998	1
GSE360_{C}TRL_{V}S_{L}_DONOVANI_{D}C_{D} … (75)	AML	0.0403	0.999	1	0.729	1	1
GSE22886_{N}AIVE_{C}D4_{T}CELL_{V}S_{N}E … (92)	AML	0.0403	0.999	1	0.758	1	1
GSE3982_{C}TRL_{V}S_{P}MA_{S}TIM_{E}OSIN … (93)	AML	0.0427	0.999	1	0.711	1	1

Results for the MSigDB C7 v5.0 collection and the Armstrong et al. [34] leukemia gene expression data (1910 total gene sets after size-based filtering). Significant q-values are marked in bold with a *before the gene set name

**Table 7:** p53 gene expression results

Gene set	Direction	GSE	Unweighted	MLRT	SGSE	TWT	MPDT
		*p*-value	*q*-value	wFDR	wFDR	wFDR	wFDR
^∗^ P53_DN.V1_UP (10)	MUT	5.21e-09	**9.79e-07**	**1.43e-07**	**6.53e-07**	**1.31e-06**	**1.09e-05**
^∗^ P53_DN.V1_DN (14)	WT	8.67e-07	**8.15e-05**	**1.19e-05**	**9.56e-05**	**6.96e-06**	**1.09e-05**
RB_P130_DN.V1_DN (123)	MUT	0.0342	0.914	1	0.505	1	1
BCAT.100_UP.V1_UP (103)	MUT	0.0385	0.914	1	0.517	1	1
^∗^ VEGF_A_UP.V1_DN (114)	MUT	0.0432	0.914	0.315	0.505	**0.231**	1
EGFR_UP.V1_DN (136)	MUT	0.046	0.914	0.315	0.505	1	0.989
RB_DN.V1_DN (110)	MUT	0.056	0.914	1	0.505	1	1
^∗^ CORDENONSI_YAP_CONSERVED_SIGNA… (124)	MUT	0.0593	0.914	0.324	0.505	**0.238**	**0.208**
RAF_UP.V1_UP (98)	MUT	0.0597	0.914	0.873	0.505	1	1
SRC_UP.V1_UP (98)	WT	0.0604	0.914	1	1	1	1
^∗^ RPS14_DN.V1_DN (98)	MUT	0.0651	0.914	1	0.734	1	**0.208**
HOXA9_DN.V1_DN (105)	MUT	0.0685	0.914	1	0.505	1	1
CSR_EARLY_UP.V1_UP (159)	MUT	0.0719	0.914	0.958	0.505	1	1
TBK1.DF_DN (150)	MUT	0.0797	0.914	1	0.56	1	1
EGFR_UP.V1_UP (17)	MUT	0.0832	0.914	0.332	0.517	1	1
MEK_UP.V1_UP (9)	MUT	0.085	0.914	0.332	0.505	1	1
*GCNP_SHH_UP_EARLY.V1_UP (170)	MUT	0.0875	0.914	0.958	1	**0.281**	1
ESC_J1_UP_EARLY.V1_DN (156)	MUT	0.0899	0.914	1	0.734	1	1
ERB2_UP.V1_UP (170)	MUT	0.103	0.914	1	0.56	1	1
KRAS.300_UP.V1_UP (155)	WT	0.108	0.914	1	0.994	1	1
BRCA1_DN.V1_UP (64)	WT	0.125	0.914	1	1	1	1
AKT_UP.V1_UP (87)	MUT	0.127	0.914	1	0.821	1	1
ERB2_UP.V1_DN (90)	MUT	0.13	0.914	0.444	0.56	1	1
STK33_SKM_DN (97)	MUT	0.139	0.914	1	0.56	1	1
ALK_DN.V1_UP (100)	WT	0.146	0.914	1	1	1	1

Results for the MSigDB C6 v5.0 collection and p53 [4] gene expression data (188 total gene sets after size-based filtering). Significant q-values are marked in bold with a *before the gene set name

The Additional file 1 contains similar results for all of the collections for both data sets. The Additional file 1 also contains the Spearman rank correlation values between the unsupervised gene set testing *p*-values computed by the CAMERA method and various unsupervised statistics (mean inter-gene correlation and the *p*-values from the MLRT, SGSE, TWT and MPDT methods). As seen in Tables 6 and 7 and the Additional file 1, no single method was dominant for all MSigDB collections on both data sets, however, the TWT method and SGSE method tended to provide the best overall results. The rank correlation values shown in Tables S1 and S14 of the Additional file 1 also demonstrate the general association between gene set biological activity (as represented by the CAMERA gene set test *p*-values) and the sample covariance structure of the gene set members. Section “Performance on gene expression data and MSigDB collections” below contains a more detailed discussion of the real data analysis results.

## Discussion

Gene set testing, or pathway analysis, is an important tool for analyzing and interpreting high-dimensional genomic data sets [1, 2]. Compared to approaches that use a separate test for each variable, gene set testing offers greater statistical power, superior interpretation and improved replication. Although most gene set testing methods can only be used in a supervised context, i.e., assessing the association of gene set members with an outcome variable, a number of important unsupervised use cases exist. To address the lack of effective unsupervised gene set testing methods, we recently developed the SGSE method [15] and later demonstrated the effective use of this method for screening-testing [19] in the SGSF approach [24]. Although the SGSE method was shown to be superior to existing unsupervised techniques and was able to significantly improve gene set testing power when used in the SGSF screening-testing approach, the method only supports testing against a competitive null hypothesis and is not able to effectively identify biologically relevant gene sets for a number of important use cases. To remedy the limitations of the SGSE method and other available unsupervised techniques, we developed two new unsupervised gene set tests, the Marc̆enko-Pastur Distribution Test (MPDT) and the Tracy-Widom Test (TWT). Both the MPDT and the TWT support self-contained and competitive null hypotheses and both are based on random matrix theory (RMT) findings regarding the distribution of the eigenvalues of matrices with a *white* Wishart distribution [32, 33]. As outlined in Section “Random matrix theory (RMT) benefits”, the RMT basis of both methods conveys several general benefits: improved performance on high-dimensional data, robustness to departures from normality and superior performance on small sample sizes.

Table 1 lists a set of biologically relevant population covariance matrix structures that illustrate the relative benefits of the proposed MDPT and TWT methods and existing methods such as SGSE and MLRT. These structures also formed the basis for the type I error control and power simulation designs detailed in Sections “Simulation design to assess type I error control” and “Simulation design to assess statistical power”. As shown in the type I error control results in Section “Type I error control results” and Table 4, all methods had excellent type I error control on each of the evaluated models. In contrast, the empirical power realized by each of four methods, as illustrated in Table 5, diverged significantly across each of the simulated models with each method delivering the best power for at least one model. Overall, the TWT method had the best performance, with the top power for six of the 10 cases and close to the best power for the remaining four cases. Sections “Unsupervised gene set testing for the single block model” through “Performance on gene expression data and MSigDB collections” below provide a more detailed discussion of these models and the simulation and real data analysis results.

### Unsupervised gene set testing for the single block model

For the single block model, represented by power simulation models MVN-1 thru MVN-6 detailed in Section “Simulation design to assess statistical power”, all members of the gene set have a positive pairwise correlation with almost no correlation with genes not in the set. Such a covariance structure is quite common for genomic data, e.g., microarray gene expression, and gene sets containing co-regulated genes [11] or gene sets based on the result of gene clustering [25]. For each of the first four simulations models (MVN-1 thru MVN-4), such a single block covariance structure was used in which all variances were set to 1 and all covariances were set to 0 except for the covariances between true gene set members, which were set to an equal and positive value. For these four models, the best power was generated by either the TWT method or the SGSE method with the classic MLRT method performing well on the two larger sample size models (MVN-1 and MVN-4) and the MPDT method generating average power substantially below the other methods. It is known that such a block covariance matrix structure will tend to produce a first principal component (PC) for the entire data set whose associated eigenvector has large weights of the same sign for all block members [39]. Because the SGSE method is based on the association between gene set members and the PCs of the entire data set, with weights based on the associated eigenvalue significance, it is able to perform well when the first PC effectively captures the true gene set signal. When the sample covariance matrix is computed using just the gene set members for the single block model, the first principal component can likewise be expected to have large weights of the same sign for all gene set members and a correspondingly large eigenvalue. Importantly, the largest eigenvalue of the sample covariance matrix for just the gene set members in this case can be expected to be stochastically larger than the principal eigenvalue of a sample covariance matrix for a random group of genes of the same size since, for the single block model, all covariances not between true gene set members are 0. This expected difference between the largest eigenvalues of random and non-random partitioned sample covariance matrices enabled the competitive TWT method to also perform well for the single block use case. The moderately higher average power achieved by the SGSE method relative the TWT method for lower sample sizes (MVN-2 and MVN3) may be due to the fact that the SGSE method is parametric whereas the TWT method is based on a permutation distribution. The fact that only the largest eigenvalue will likely represent the non-null gene set explains the poor performance of the MPDT method, which is based on the bulk eigenvalue distribution. The satisfactory performance of the MLRT method for the single block models was likely due to the fact that the MVN distribution of the simulated data aligned with the distributional assumptions of the MLRT test.

For single block models MVN-5 and MVN-6, the best power was provided by either the TWT method or the MPDT method. The covariance structure used for these simulations specified an elevated variance for true gene set members with all covariances set to 0. Such a covariance structure will tend to generate one PC for each variable that has an elevated variance with the loading for that variable dominating the other loadings and the eigenvalue associated with the PC similar in magnitude to the variance of the variable. The signal for the true gene set for these models was therefore spread across multiple eigenvalues of the sample covariance matrix. Because the MPDT method is based on all of eigenvalues of the partitioned sample covariance matrix, its good relative power was therefore expected for these models. Since no single PC was closely associated with the true gene set, the poor performance of the SGSE method was also expected. Because the TWT method is based on just the magnitude of the largest eigenvalue of the partitioned sample covariance matrix, it was able perform well even though the associated PC tended to represent just one gene set member. The poor power of the MLRT method in these cases was likely due to the fact that all population covariances were 0.

### Unsupervised gene set testing for the multi-block model

A multi-block covariance structure was used for model MVN-7 in which the portion of the population covariance matrix associated with the true gene set was divided into 5 disjoint 2 × 2 blocks with covariance 0.2. Multi-block covariance structures are also quite common for genomic data and biologically-based gene sets, especially large gene sets that represent processes with multiple modes of activity. Similar to the single block case, such a multi-block structure will tend to produce one PC per block [39] and will therefore distribute the signal for the true gene set across multiple eigenvalues/PCs of the sample covariance matrix. For MVN-7, the MLRT method had the best average power with the TWT and MPDT methods providing comparable performance and the SGSE method returning the worst average power. As detailed for models MVN-5 and MVN-6 above, the fact that multiple eigenvalues represent the true gene set is consistent with the good relative power of the MPDT and TWT methods and poor relative power of the SGSE method. The superior relative performance of the MLRT method for MVN-7, and the fact that the MLRT method generated much larger average power for MVN-7 than for MVN-5 or MVN-6, is likely due to the presence of non-zero population covariance values in the MVN-7 model.

The anti-correlated multi-block covariance structure used for MVN-8 was a variation of the multi-block model used for MVN-7. In this case, the portion of the covariance matrix associated with the true gene set was split into two blocks with correlations between members of the same block set to 0.1. The key difference between the multi-block structure and the anti-correlated multi-block structure used for MVN-8 is that the covariance between members of different blocks was set to -0.1 rather than 0. This type of structure also corresponds to a biologically realistic class of gene sets, e.g., a metabolic pathway that has several distinct modes of action represented by different subsets of associated genes. The covariance structure in MVN-8 leads to two PCs representing the variable group members with opposite sign loadings for the members of each block. For MVN-8, the TWT method provided the best power with the MLRT method a close second and significantly lower power for the MPDT and SGSE methods. The opposite sign loadings pose a particular problem for the SGSE method which effectively compares the mean PC loading for members of the gene set against the mean PC loading for non-gene set members. The TWT method, on the other hand, is based just on the magnitude of the largest eigenvalue so has power comparable to the single block models. The results for the MPDT and MLRT methods on MVN-8 are explained by reasoning similar to that outlined for the single block models above.

### Unsupervised gene set testing for the repeated block model

Similar to the single block and multi-block models, the repeated block model, represented by MVN-8, will tend to produce one PC per each block that has large and equal signed loadings for all block members. Such a model can be expected when there are multiple independent gene sets associated with a specific data set. Although every gene set in this case has a population covariance matrix with equal and non-zero covariance values, the gene sets are significant relative to *H*_0_ (8) in the main manuscript since a random partition of the sample covariance matrix of the same size as the gene sets will tend to include zero covariance values as well due to the zero covariance between members of different gene sets For model MVN-9, the TWT method had substantially higher power than all other methods. In this case, the population covariance matrix was divided into one block per disjoint gene set. For the SGSE method, the poor power is explained by the fact that each gene set will only be associated with one PC yet multiple PCs will have significant eigenvalues so, when the association measures are combined for all PCs, the gene set will not appear enriched relative to a competitive null hypothesis. The poor power of the MPDT and MLRT methods relative to the TWT method is likely due to the fact that MPDT and MLRT consider all of the eigenvalues of the partitioned sample covariance matrix whereas the TWT method is based on just the largest eigenvalue.

### Unsupervised gene set testing for non-normal data

For many types of genomic data, such as the genotypic data collected by genome-wide association studies, the measured values of genomic variables are not normally distributed. For the case of single nucleotide polymorphisms (SNPs) specified using additive coding, a data model similar to Binom-1 can be expected. For this model, the TWT method had the best average power followed closely by the SGSE method. The superior performance of the TWT and SGSE methods relative to the MPDT and MLRT methods follows from the universality properties of the Tracy-Widom distribution, i.e., the Tracy-Widom distribution of the scaled and centered largest eigenvalue is known to be robust to departures from normality for **x** [38].

### Performance on gene expression data and MSigDB collections

To assess the practical utility of the TWT and the MPDT methods, a wFDR analysis (detailed in Section “Real data analysis design”) was performed on two real gene expression data sets relative to MSigDB gene set collections. As detailed in Section “Real data results” above and the Additional file 1, the results on the p53 and leukemia data sets mirrored the results on the simulation examples. Specifically, the relative ranking of the four methods varied considerably across the MSigDB collections and two data sets with the TWT and SGSE methods delivering the best overall performance. As seen in Table 6, the eight most significantly enriched C7 gene sets relative to AML vs. ALL status represent the differential expression of different types of white blood cells (primarily lymphoid vs. myeloid cells) and are therefore biologically consistent with the phenotype. Although these gene sets have enrichment *p*-values that are significant prior to MHC, after controlling the FDR for the family of all 1910 analyzed C7 gene sets, none have significant *q*-values. When a wFDR approach is taken using weights based on the *p*-values from the TWT test, three of the top eight gene sets had significant *q*-values (these are marked in bold in Table 6 with a * prefixing the gene set name). When weights were based on either the MLRT, SGSE or MPDT method, no significant *q*-values were generated.

For the p53 gene expression data and C6 collection, the impact of weighting was less pronounced. As seen in Table 7, the two most significantly enriched C6 gene sets relative to p53 mutated status represent either up-regulated or down-regulated genes in the NCI-60 panel of cell lines with mutated p53. In this case, both gene sets had significant *q*-values without any weighting and when *p*-values were weighted using any of the unsupervised gene set tests. However, the use of the TWT and MPDT methods to generate weights also produced marginally significant *q*-values of less than 0.3 for several other biologically plausible gene sets for the p53 data. Similar to the leukemia results, these *q*-values are marked in bold with * prefixing the gene set names.

Tables S1 and S14 in the Additional file 1 show the overall association between the supervised gene set test *p*-values generated by the CAMERA method and either the mean inter-gene correlation among gene set members (as estimated by CAMERA) or the unsupervised *p*-values generated by the MLRT, SGSE, TWT and MPDT methods. These tables demonstrate the general association between the departure of gene set members from an identity covariance structure and biological activity as represented by the supervised gene set *p*-values. As seen in these tables, the *p*-values generated by the TWT and SGSE methods have the largest correlation with the supervised *p*-values for both data sets across the different MSigDB gene set collections. In contrast, the mean inter-gene correlation estimated by the CAMERA method, while still associated with the supervised *p*-values, is a comparatively poor predictor of gene set biological activity.

## Conclusions

The TWT and MPDT methods represent important methodological advances for unsupervised gene set testing. These new methods support both self-contained and competitive null hypotheses and provide performance superior to existing approaches, such as the SGSE and MLRT methods, on a set of biologically important data structures. The TWT method provides good power across most expected models and is clearly the best choice for non-normal data (e.g., model Binom-1), an anti-correlated multi-block structure (e.g., model MVN-7) or a repeated block structure (e.g., model MVN-8). If a single block structure can be expected with standardized variance (i.e., all members of the gene set have a positive pairwise correlation with almost no correlation with genes not in the set and variance of 1) and the number of samples is small relative to the size of the gene set (i.e., *n*/*p*≤5), as represented by models MVN-2 and MVN-3, then the SGSE method is the best choice. If variance of each gene in the set is large relative to genes not in the set (as represented by model MVN-6), then the MPDT method can provide the best results. For multi-block data (e.g., model MVN-7) or data following a single block structure with a large correlation between gene set members (e.g., MVN-4), the best results are generated by the classic MLRT method.

Important directions for future research include the assessment of a broader range of biologically relevant covariance structures, the exploration of other classes of non-normal data, and the use of the TWT and MPDT methods to make novel biological findings via *p*-value weighting.

## Additional file

Additional file 1 Supplementary results for leukemia and p53 gene expression examples. (168 KB PDF)

## Abbreviations

ALL: Acute lymphoblastic leukemia
AML: Acute myeloid leukemia
CAMERA: Correlation adjusted mean rank gene set test
FDR: False discovery rate
GO: Gene ontology
MHC: Multiple hypothesis correction
MLRT: Modified likelihood ratio criterion test
MPDT: Marc̆enko-Pastur distribution test
MSigDB: Molecular signatures database
MVN: Multivariate normal
PC: Principal component
PCGSE: Principal component gene set enrichment
RMT: Random matrix theory
SGSE: Spectral gene set enrichment
SNP: Single nucleotide polymorphism
TWT: Tracy-Widom Test
wFDR: Weighted false discovery rate
