# Supplementary material for: Unsupervised gene set testing based on random matrix theory
Source: BMC Bioinformatics. 2016 Nov 4;17:442. doi: 10.1186/s12859-016-1299-8 (PMC5096314; doi:10.1186/s12859-016-1299-8)
Supplement: Additional file 1 — Supplementary results for leukemia and p53 gene expression examples. (168 KB PDF) [file 12859_2016_1299_MOESM1_ESM.pdf]

Unsupervised gene set testing based on random matrix theory
Supplemental Material

H. Robert Frost and Christopher I. Amos

Contents

1 Results for v5.0 MSigDB collections and leukemia gene expression data **2**

2 Results for v5.0 MSigDB collections and p53 gene expression data **10**

List of Tables

S1 Spearman rank correlation values between CAMERA supervised gene set test p-values and either the mean inter-gene correlation or unsupervised gene set test p-values. 3
S2 Results for MSigDB c1.all.v5.0 collection (281 total gene sets after size-based filtering) . . . 3
S3 Results for MSigDB c2.cgp.v5.0 collection (2967 total gene sets after size-based filtering) . . 4
S4 Results for MSigDB c2.cp.v5.0 collection (1301 total gene sets after size-based filtering) . . 4
S5 Results for MSigDB c3.mir.v5.0 collection (201 total gene sets after size-based filtering) . . 5
S6 Results for MSigDB c3.tft.v5.0 collection (565 total gene sets after size-based filtering) . . . 5
S7 Results for MSigDB c4.cgn.v5.0 collection (401 total gene sets after size-based filtering) . . 6
S8 Results for MSigDB c4.cm.v5.0 collection (376 total gene sets after size-based filtering) . . . 6
S9 Results for MSigDB c5.bp.v5.0 collection (743 total gene sets after size-based filtering) . . . 7
S10 Results for MSigDB c5.cc.v5.0 collection (197 total gene sets after size-based filtering) . . . 7
S11 Results for MSigDB c5.mf.v5.0 collection (370 total gene sets after size-based filtering) . . . 8
S12 Results for MSigDB c6.all.v5.0 collection (188 total gene sets after size-based filtering) . . . 8
S13 Results for MSigDB c7.all.v5.0 collection (1910 total gene sets after size-based filtering) . . 9
S14 Spearman rank correlation values between CAMERA supervised gene set test p-values and either the mean inter-gene correlation or unsupervised gene set test p-values. 11
S15 Results for MSigDB c1.all.v5.0 collection (281 total gene sets after size-based filtering) . . . 11
S16 Results for MSigDB c2.cgp.v5.0 collection (2966 total gene sets after size-based filtering) . . 12
S17 Results for MSigDB c2.cp.v5.0 collection (1300 total gene sets after size-based filtering) . . 12
S18 Results for MSigDB c3.mir.v5.0 collection (201 total gene sets after size-based filtering) . . 13
S19 Results for MSigDB c3.tft.v5.0 collection (565 total gene sets after size-based filtering) . . . 13
S20 Results for MSigDB c4.cgn.v5.0 collection (401 total gene sets after size-based filtering) . . 14
S21 Results for MSigDB c4.cm.v5.0 collection (375 total gene sets after size-based filtering) . . . 14
S22 Results for MSigDB c5.bp.v5.0 collection (743 total gene sets after size-based filtering) . . . 15
S23 Results for MSigDB c5.cc.v5.0 collection (197 total gene sets after size-based filtering) . . . 15
S24 Results for MSigDB c5.mf.v5.0 collection (370 total gene sets after size-based filtering) . . . 16
S25 Results for MSigDB c6.all.v5.0 collection (188 total gene sets after size-based filtering) . . . 16
S26 Results for MSigDB c7.all.v5.0 collection (1910 total gene sets after size-based filtering) . . 17

1 Results for v5.0 MSigDB collections and leukemia gene expression data

This section contains results for the evaluation detailed in Section 2.6.4 of the main manuscript for all analyzed v5.0 MSigDB collections and the Armstrong et al. leukemia gene expression data set.

Table S1 contains the Spearman rank correlation values between the supervised gene set testing p-values (as computed by the CAMERA method) and the unsupervised gene set testing p-values (as computed by the MLRT, TWT, SGSE or MPDT methods) for each MSigDB collection relative to the AML vs. ALL phenotype.

Tables S2-S13 below display the 25 gene sets from each MSigDB collection with the most significant p-value from a supervised gene set test relative to the AML vs. ALL phenotype as computed using the CAMERA method with configuration specified in Section 2.6.5 of the main manuscript. The first column in the table contains the gene set name with the number of genes in the set in parentheses. The second column lists the direction of enrichment, the third column the enrichment significance as computed via CAMERA and the forth column the false discovery rate q-value when all considering all gene sets in the collection as the family of hypotheses. Columns five through eight display the results of a weighted FDR analysis, as detailed in Section 2.6.4 of the main manuscript, using each of the evaluated unsupervised gene set testing methods to compute the weight applied to the CAMERA p-value.

MSigDB Collection	Inter-gene correl.	MLRT	SGSE	TWT	MPDT
c1.all.v5.0	-0.0363	0.0478	0.441	0.177	0.0574
c2.cgp.v5.0	0.0403	0.0732	0.455	0.137	-0.00833
c2.cp.v5.0	0.23	0.265	0.4	0.401	0.174
c3.mir.v5.0	0.0881	0.0825	0.401	0.407	0.0154
c3.tft.v5.0	0.0591	0.076	0.492	0.224	-0.0592
c4.cgn.v5.0	0.203	0.28	0.092	0.368	-0.103
c4.cm.v5.0	0.00957	0.0853	0.341	0.16	-0.0101
c5.bp.v5.0	0.057	0.154	0.414	0.271	0.0547
c5.cc.v5.0	0.0555	0.187	0.284	0.333	0.0604
c5.mf.v5.0	0.173	0.342	0.474	0.415	0.22
c6.all.v5.0	-0.177	-0.159	0.584	0.0505	-0.118
c7.all.v5.0	0.188	0.102	0.442	0.342	-0.148

Table S1: Spearman rank correlation values between CAMERA supervised gene set test p-values and either the mean inter-gene correlation or unsupervised gene set test p-values.

Gene set	Direction	GSE p-value	Unweighted q-value	MLRT wFDR	SGSE wFDR	TWT wFDR	MPDT wFDR
chr1q12 (28)	AML	0.0172	0.998	1	1	1	1
chr14q13 (27)	ALL	0.0475	0.998	1	1	1	1
chr21q21 (51)	ALL	0.0561	0.998	1	1	1	1
chr10p14 (5)	ALL	0.0561	0.998	1	1	1	1
chr1q23 (15)	AML	0.0805	0.998	1	1	1	1
chr12q11 (38)	AML	0.0835	0.998	1	1	1	1
chr2q35 (7)	AML	0.107	0.998	1	1	1	1
chr15q25 (18)	AML	0.108	0.998	1	1	1	1
chr7p14 (45)	AML	0.112	0.998	1	1	1	1
chr16q13 (14)	AML	0.114	0.998	1	1	1	1
chr2p16 (17)	ALL	0.12	0.998	1	1	1	1
chr12q12 (5)	AML	0.129	0.998	1	1	1	1
chr11q24 (10)	AML	0.134	0.998	1	1	1	1
chr15q23 (6)	AML	0.139	0.998	1	1	1	1
chr5q33 (15)	AML	0.141	0.998	1	1	1	1
chr4p14 (5)	ALL	0.147	0.998	1	1	1	1
chr6p22 (13)	ALL	0.162	0.998	0.828	1	1	1
chr1q43 (14)	ALL	0.165	0.998	1	1	1	1
chr17q25 (39)	AML	0.172	0.998	1	1	1	1
chr2p21 (26)	ALL	0.181	0.998	1	1	1	1
chr5p15 (49)	ALL	0.183	0.998	1	1	1	1
chr2p13 (8)	AML	0.184	0.998	1	1	1	1
chr12q (25)	ALL	0.187	0.998	1	1	1	1
chr6q25 (7)	ALL	0.188	0.998	1	1	1	1
chr9q32 (20)	AML	0.196	0.998	1	1	1	1

Table S2: Results for MSigDB c1.all.v5.0 collection (281 total gene sets after size-based filtering)

Gene set	Direction	GSE p-value	Unweighted q-value	MLRT wFDR	SGSE wFDR	TWT wFDR	MPDT wFDR
HADDAD_B_LYMPHOCYTE_PROGENITOR (16)	ALL	0.00065	0.888	0.0723	0.396	0.067	0.805
GOLUB_ALL_VS_AML_DN (28)	AML	0.000971	0.888	0.673	0.372	0.067	0.805
TONG_INTERACT_WITH_PTTG1 (14)	AML	0.0013	0.888	0.673	0.372	0.581	0.805
GOLUB_ALL_VS_AML_UP (18)	ALL	0.00173	0.888	0.673	0.41	0.0795	0.805
NAKAJIMA_MAST_CELL (53)	AML	0.00244	0.888	0.673	0.41	0.581	0.805
VERRECCHIA_EARLY_RESPONSE_TO_T... (31)	AML	0.00247	0.888	0.725	0.396	0.891	1
GUENTHER_GROWTH_SPHERICAL_VS_A... (35)	AML	0.00274	0.888	0.687	0.396	0.581	0.805
VERRECCHIA_RESPONSE_TO_TGFB1_C... (37)	AML	0.00275	0.888	0.72	0.396	1	1
CHEOK_RESPONSE_TO_HD_MTX_UP (6)	AML	0.0033	0.888	0.673	0.396	0.581	0.805
ALONSO_METASTASIS_NEURAL_UP (151)	AML	0.00385	0.888	0.687	0.417	0.644	0.805
HOLLEMAN_PREDNISOLONE_RESISTAN... (129)	AML	0.00446	0.888	0.725	0.416	0.581	1
HUPER_BREAST_BASAL_VS_LUMINAL_... (71)	AML	0.00475	0.888	0.695	0.467	1	0.805
OXFORD_RALB_TARGETS_UP (16)	AML	0.00487	0.888	1	0.467	1	1
KLEIN_PRIMARY_EFFUSION_LYMPHOM... (6)	ALL	0.00539	0.888	0.673	0.702	0.581	0.805
SHI_SPARC_TARGETS_DN (17)	AML	0.00689	0.888	1	0.559	1	1
WEST_ADRENOCORTICAL_CARCINOMA_... (9)	ALL	0.00703	0.888	0.695	0.56	0.581	0.805
LEE_LIVER_CANCER_E2F1_UP (11)	AML	0.00724	0.888	0.783	0.454	1	1
HU_ANGIOGENESIS_UP (18)	AML	0.00865	0.888	1	0.467	1	1
KIM_ALL_DISORDERS_CALB1_CORR_D... (89)	AML	0.00966	0.888	1	0.5	0.952	1
TONKS_TARGETS_OF_RUNX1_RUNX1T1... (82)	AML	0.0098	0.888	1	0.467	1	0.805
VERNOCHET_ADIPOGENESIS (134)	AML	0.00983	0.888	0.72	0.559	0.606	0.875
SABATES_COLORECTAL_ADENOMA_UP (159)	AML	0.0109	0.888	0.972	0.559	1	1
WANG_BARRETTS_ESOPHAGUS_AND_ES... (49)	AML	0.0111	0.888	1	0.467	0.603	1
HILLION_HMGA1B_TARGETS (81)	AML	0.0129	0.888	0.826	0.416	1	0.979
DALESSIO_TSA_RESPONSE (197)	AML	0.0133	0.888	1	0.559	1	1

Table S3: Results for MSigDB c2.cgp.v5.0 collection (2967 total gene sets after size-based filtering)

Gene set	Direction	GSE p-value	Unweighted q-value	MLRT wFDR	SGSE wFDR	TWT wFDR	MPDT wFDR
BIOCARTA_DC_PATHWAY (49)	AML	0.00793	0.967	0.825	0.779	0.89	1
REACTOME_CELL_SURFACE_INTERACT... (26)	AML	0.0104	0.967	1	0.779	1	0.651
REACTOME_HYALURONAN_UPTAKE_AND... (20)	AML	0.0108	0.967	0.738	0.779	0.982	0.988
PID_INTEGRIN2_PATHWAY (12)	AML	0.0137	0.967	0.599	0.779	0.493	0.936
PID_UPA_UPAR_PATHWAY (29)	AML	0.0137	0.967	0.758	0.779	0.738	1
SA_MMP_CYTOKINE_CONNECTION (22)	AML	0.014	0.967	1	0.779	0.89	1
REACTOME_REGULATION_OF_INSULIN... (12)	AML	0.016	0.967	0.997	0.779	1	1
PID_INTEGRIN_CS_PATHWAY (34)	AML	0.0185	0.967	1	0.779	1	1
PID_INTEGRIN5_PATHWAY (14)	AML	0.019	0.967	1	0.779	1	1
BIOCARTA_BLYMPHOCYTE_PATHWAY (11)	AML	0.0191	0.967	1	0.779	1	1
PID_ERB_GENOMIC_PATHWAY (35)	ALL	0.0197	0.967	1	0.779	0.723	1
REACTOME_HS_GAG_DEGRADATION (80)	AML	0.0208	0.967	1	0.779	1	1
REACTOME_CHONDROITIN_SULFATE_B... (112)	AML	0.022	0.967	1	0.779	1	1
BIOCARTA_LAIR_PATHWAY (62)	AML	0.0221	0.967	1	0.779	1	1
REACTOME_TELOMERE_MAINTENANCE (25)	ALL	0.0249	0.967	0.44	0.779	0.407	0.271
KEGG_ARACHIDONIC_ACID_METABOLI... (25)	AML	0.0258	0.967	1	0.779	1	1
KEGG_RENIN_ANGIOTENSIN_SYSTEM (22)	AML	0.0258	0.967	1	0.779	1	1
PID_SYNDECAN_1_PATHWAY (36)	AML	0.0264	0.967	1	0.84	1	1
REACTOME_ANTIGEN_ACTIVATES_B_C... (7)	ALL	0.0274	0.967	1	0.779	0.561	1
REACTOME_MEIOTIC_SYNAPSIS (21)	ALL	0.0299	0.967	0.322	0.779	0.508	0.271
REACTOME_PHASE1_FUNCTIONALIZAT... (40)	AML	0.0301	0.967	0.806	0.815	0.738	1
BIOCARTA_DNAFRAGMENT_PATHWAY (19)	ALL	0.031	0.967	1	0.779	1	1
PID_RXR_VDR_PATHWAY (34)	AML	0.0313	0.967	1	0.779	1	1
BIOCARTA_CYTOKINE_PATHWAY (17)	AML	0.0346	0.967	1	0.779	1	1
REACTOME_COLLAGEN_FORMATION (28)	AML	0.0347	0.967	1	0.779	1	1

Table S4: Results for MSigDB c2.cp.v5.0 collection (1301 total gene sets after size-based filtering)

Gene set	Direction	GSE p-value	Unweighted q-value	MLRT wFDR	SGSE wFDR	TWT wFDR	MPDT wFDR
ACCGAGC,MIR-423 (161)	AML	0.0501	0.993	1	1	1	1
GGCACAT,MIR-455 (180)	ALL	0.151	0.993	1	1	1	1
AGCGCAG,MIR-191 (141)	ALL	0.152	0.993	1	1	1	1
CGGTGTG,MIR-220 (10)	AML	0.171	0.993	1	1	1	1
ATCTTGC,MIR-31 (145)	ALL	0.171	0.993	1	1	1	1
ACCATTT,MIR-522 (140)	ALL	0.185	0.993	1	1	1	1
AGTCTTA,MIR-499 (13)	ALL	0.19	0.993	1	1	1	1
AAGTCCA,MIR-422B,MIR-422A (25)	ALL	0.19	0.993	1	1	1	1
GACTGTT,MIR-212,MIR-132 (199)	ALL	0.191	0.993	1	1	1	1
GGGATGC,MIR-324-5P (8)	ALL	0.192	0.993	1	1	1	1
TAGGTCA,MIR-192,MIR-215 (159)	ALL	0.193	0.993	1	1	1	1
AAAGGAT,MIR-501 (34)	ALL	0.207	0.993	1	1	1	1
GTAGGCA,MIR-189 (114)	ALL	0.211	0.993	1	1	1	1
ACATTCC,MIR-1,MIR-206 (172)	ALL	0.211	0.993	1	1	1	1
CACGTTT,MIR-302A (100)	ALL	0.218	0.993	1	1	1	1
GCACCTT,MIR-18A,MIR-18B (154)	ALL	0.222	0.993	1	1	1	1
TTCCGTT,MIR-191 (155)	AML	0.224	0.993	1	1	1	1
AATGGAG,MIR-136 (40)	ALL	0.254	0.993	1	1	1	1
GCTCTTG,MIR-335 (33)	ALL	0.274	0.993	1	1	1	1
AACTGAC,MIR-223 (180)	ALL	0.286	0.993	1	1	1	1
TCTCTCC,MIR-185 (28)	AML	0.301	0.993	1	1	1	1
AGGAGTG,MIR-483 (27)	ALL	0.311	0.993	1	1	1	1
TACGGGT,MIR-99A,MIR-100,MIR-99... (62)	ALL	0.313	0.993	1	1	1	1
TCCAGAT,MIR-516-5P (7)	ALL	0.313	0.993	1	1	1	1
ATTACAT,MIR-380-3P (68)	ALL	0.314	0.993	1	1	1	1

Table S5: Results for MSigDB c3.mir.v5.0 collection (201 total gene sets after size-based filtering)

Gene set	Direction	GSE p-value	Unweighted q-value	MLRT wFDR	SGSE wFDR	TWT wFDR	MPDT wFDR
V$SRF_01 (113)	AML	0.0101	0.997	1	0.499	1	1
V$SRF_Q5_01 (198)	AML	0.0646	0.997	1	1	1	1
KCCGNSWTTT_UNKNOWN (121)	ALL	0.0871	0.997	0.822	1	1	1
CTGRYYYNATT_UNKNOWN (133)	AML	0.104	0.997	1	1	1	1
CCAWWNAAGG_V$SRF_Q4 (127)	AML	0.105	0.997	1	1	1	1
V$NRSF_01 (130)	AML	0.133	0.997	1	1	1	1
V$SRF_Q6 (76)	AML	0.134	0.997	1	1	1	1
ACAWNRNSRCGG_UNKNOWN (128)	ALL	0.146	0.997	1	1	1	1
CCANNAGRKGGC_UNKNOWN (123)	AML	0.157	0.997	1	1	1	1
V$SRF_C (15)	AML	0.16	0.997	1	1	1	1
V$NFMUE1_Q6 (148)	ALL	0.175	0.997	1	1	1	1
GATAAGR_V$GATA_C (22)	AML	0.177	0.997	1	1	1	1
TGTYNNNNNRGCARM_UNKNOWN (44)	AML	0.188	0.997	1	1	1	1
V$AP1_01 (114)	AML	0.189	0.997	1	1	1	1
V$NFKAPPAB_01 (132)	AML	0.19	0.997	1	1	1	1
V$SRF_Q4 (124)	AML	0.195	0.997	1	1	1	1
V$YY1_02 (61)	ALL	0.196	0.997	1	1	1	1
TTTNNANAGCYR_UNKNOWN (132)	ALL	0.198	0.997	1	1	1	1
CCAWNWWNNNGGC_UNKNOWN (151)	ALL	0.2	0.997	1	1	1	1
CAGNWMCNNNGAC_UNKNOWN (130)	AML	0.204	0.997	1	1	1	1
V$GATA3_01 (84)	AML	0.215	0.997	1	1	1	1
V$YY1_Q6 (133)	ALL	0.225	0.997	1	1	1	1
TMTCGCGANR_UNKNOWN (137)	ALL	0.228	0.997	1	1	1	1
V$GATA1_04 (115)	AML	0.229	0.997	1	1	1	1
V$LXR_Q3 (117)	AML	0.23	0.997	1	1	1	1

Table S6: Results for MSigDB c3.tft.v5.0 collection (565 total gene sets after size-based filtering)

Gene set	Direction	GSE p-value	Unweighted q-value	MLRT wFDR	SGSE wFDR	TWT wFDR	MPDT wFDR
GNF2_IGFBP1 (156)	AML	0.0189	0.425	0.492	0.393	0.446	0.433
GNF2_EGFR (68)	AML	0.0193	0.425	0.538	0.412	0.491	0.586
GNF2_CDKN1C (178)	AML	0.0351	0.425	0.894	0.917	0.786	1
MORF_FLT1 (154)	AML	0.0379	0.425	0.394	0.393	0.379	1
GCM_MYCL1 (40)	AML	0.0399	0.425	1	0.393	0.901	1
MORF_THRA (158)	AML	0.042	0.425	0.716	0.393	0.379	0.384
MORF_PRKACA (77)	AML	0.0431	0.425	0.394	0.393	0.379	1
MORF_BCL2 (46)	AML	0.0454	0.425	0.394	0.393	0.379	1
MORF_SMC1L1 (135)	ALL	0.046	0.425	0.394	0.412	0.379	0.384
MORF_RAB11A (53)	ALL	0.0475	0.425	0.643	0.442	0.379	0.384
MORF_STK17A (70)	AML	0.0497	0.425	0.394	0.393	0.379	1
MORF_TFDP2 (96)	AML	0.0499	0.425	0.394	0.393	0.379	1
MORF_JAG1 (77)	AML	0.05	0.425	0.394	0.393	0.379	1
GNF2_CD14 (56)	AML	0.05	0.425	0.835	0.393	0.677	0.708
MORF_NF1 (183)	AML	0.0509	0.425	0.394	0.393	0.379	1
MORF_CDH4 (56)	AML	0.0512	0.425	0.394	0.393	0.379	1
GNF2_TM4SF2 (48)	AML	0.0512	0.425	1	0.498	1	1
MORF_SP3 (142)	ALL	0.0514	0.425	0.394	0.478	0.379	0.384
MORF_ARAF1 (195)	AML	0.0518	0.425	0.394	0.393	0.379	1
MORF_RAGE (49)	AML	0.0528	0.425	0.394	0.393	0.379	1
MORF_CD8A (192)	AML	0.0533	0.425	0.394	0.393	0.677	1
GNF2_CD1D (135)	AML	0.0537	0.425	0.882	0.393	0.837	0.384
MORF_LTK (74)	AML	0.054	0.425	0.394	0.393	0.379	1
MORF_MYC (68)	AML	0.0542	0.425	0.394	0.393	0.379	1
MORF_MSH2 (148)	ALL	0.0559	0.425	0.394	0.412	0.379	0.384

Table S7: Results for MSigDB c4.cgn.v5.0 collection (401 total gene sets after size-based filtering)

Gene set	Direction	GSE p-value	Unweighted q-value	MLRT wFDR	SGSE wFDR	TWT wFDR	MPDT wFDR
MODULE_14 (17)	AML	0.000263	0.0895	0.409	0.0315	0.234	0.516
MODULE_178 (188)	AML	0.000476	0.0895	0.409	0.0315	0.234	0.516
MODULE_362 (71)	AML	0.000828	0.104	0.409	0.0368	0.324	0.516
MODULE_444 (15)	AML	0.00155	0.145	0.503	0.0745	0.355	0.516
MODULE_275 (71)	AML	0.00434	0.326	0.62	0.189	0.51	0.784
MODULE_516 (10)	AML	0.00535	0.335	0.62	0.189	0.53	0.647
MODULE_562 (38)	AML	0.00959	0.484	0.746	0.205	1	1
MODULE_128 (13)	AML	0.0135	0.484	0.737	0.205	1	0.647
MODULE_412 (63)	AML	0.0135	0.484	0.696	0.254	0.795	1
MODULE_170 (31)	AML	0.0136	0.484	0.737	0.205	1	0.647
MODULE_79 (26)	AML	0.0141	0.484	0.737	0.205	1	0.647
MODULE_204 (181)	ALL	0.0193	0.523	0.62	0.26	0.361	1
MODULE_379 (13)	AML	0.0227	0.523	0.746	0.205	0.234	1
MODULE_242 (115)	AML	0.0245	0.523	0.746	0.205	0.234	1
MODULE_108 (8)	AML	0.0246	0.523	0.737	0.254	1	1
MODULE_426 (77)	AML	0.0268	0.523	1	0.254	1	1
MODULE_189 (21)	ALL	0.0269	0.523	0.696	0.487	0.561	0.647
MODULE_247 (91)	AML	0.0277	0.523	0.696	0.254	1	0.779
MODULE_210 (167)	AML	0.0318	0.523	1	0.301	1	1
MODULE_326 (150)	AML	0.0332	0.523	0.737	0.315	1	1
MODULE_274 (105)	AML	0.0359	0.523	0.86	0.26	1	1
MODULE_277 (13)	ALL	0.0364	0.523	0.746	0.419	0.561	0.786
MODULE_63 (35)	AML	0.0365	0.523	0.86	0.365	1	1
MODULE_127 (173)	ALL	0.038	0.523	0.737	0.627	0.848	0.786
MODULE_552 (12)	ALL	0.0392	0.523	0.696	0.495	0.606	0.729

Table S8: Results for MSigDB c4.cm.v5.0 collection (376 total gene sets after size-based filtering)

Gene set	Direction	GSE p-value	Unweighted q-value	MLRT wFDR	SGSE wFDR	TWT wFDR	MPDT wFDR
ADENYLATE_CYCLASE_ACTIVATION (5)	AML	0.00571	0.97	0.922	0.5	0.703	1
ACUTE_INFLAMMATORY_RESPONSE (175)	AML	0.012	0.97	0.922	0.5	0.857	1
PROTEOGLYCAN_METABOLIC_PROCESS (9)	AML	0.0142	0.97	0.922	0.5	0.857	1
REGULATION_OF_PROTEIN_IMPORT_I... (85)	AML	0.0143	0.97	0.922	0.5	0.857	1
G_PROTEIN_SIGNALING_ADENYLATE_... (10)	AML	0.015	0.97	0.922	0.5	0.857	1
RESPONSE_TO_TOXIN (7)	AML	0.0168	0.97	0.922	0.5	0.911	1
PROTEOGLYCAN_BIOSYNTHETIC_PROC... (24)	AML	0.0191	0.97	0.922	0.5	0.857	1
REGULATION_OF_HOMEOSTATIC_PROC... (10)	AML	0.0191	0.97	0.939	0.5	1	1
GLUTAMATE_SIGNALING_PATHWAY (94)	AML	0.0199	0.97	0.922	0.5	0.857	1
ICOSANOID_METABOLIC_PROCESS (21)	AML	0.0218	0.97	0.922	0.5	0.975	1
BONE_REMODELING (35)	AML	0.0219	0.97	0.922	0.5	0.857	1
SULFUR_COMPOUND_BIOSYNTHETIC_P... (71)	AML	0.0221	0.97	0.922	0.5	0.857	1
SENSORY_ORGAN_DEVELOPMENT (18)	AML	0.0287	0.97	0.922	0.563	0.911	1
G_PROTEIN_SIGNALING_COUPLED_TO... (15)	AML	0.0298	0.97	0.922	0.563	0.857	1
VACUOLE_ORGANIZATION_AND_BIOGE... (12)	AML	0.0312	0.97	0.922	0.5	0.911	1
PYRIMIDINE_NUCLEOTIDE_METABOLI... (41)	ALL	0.0319	0.97	0.922	0.563	0.857	1
G_PROTEIN_SIGNALING_COUPLED_TO... (5)	AML	0.0328	0.97	0.922	0.5	0.857	1
CYCLIC_NUCLEOTIDE_MEDIATED_SIG... (18)	AML	0.0336	0.97	0.922	0.563	0.857	1
LOCOMOTORY_BEHAVIOR (23)	AML	0.0337	0.97	0.922	0.5	0.857	1
CAMP_MEDIATED_SIGNALING (22)	AML	0.0383	0.97	0.922	0.563	0.857	1
STRIATED_MUSCLE_CONTRACTION_GO... (126)	AML	0.039	0.97	1	0.563	1	1
LEUKOCYTE_MIGRATION (9)	AML	0.0395	0.97	0.922	0.563	0.939	1
G_PROTEIN_SIGNALING_ADENYLATE_... (8)	AML	0.0396	0.97	0.922	0.5	0.911	1
LEUKOCYTE_CHEMOTAXIS (19)	AML	0.0402	0.97	0.922	0.583	0.911	1
TISSUE_REMODELING (38)	AML	0.0407	0.97	0.922	0.563	0.857	1

Table S9: Results for MSigDB c5.bp.v5.0 collection (743 total gene sets after size-based filtering)

Gene set	Direction	GSE p-value	Unweighted q-value	MLRT wFDR	SGSE wFDR	TWT wFDR	MPDT wFDR
SYNAPTIC_VESICLE (167)	AML	0.000957	0.189	0.376	0.0442	0.323	0.926
CLATHRIN_COATED_VESICLE (6)	AML	0.0136	0.718	1	0.422	1	1
SECRETORY_GRANULE (11)	AML	0.0291	0.718	1	0.7	1	1
NUCLEAR_BODY (16)	ALL	0.0342	0.718	1	0.7	1	1
INTERCELLULAR_JUNCTION (27)	AML	0.0646	0.718	1	0.7	1	1
EXTERNAL_SIDE_OF_PLASMA_MEMBRA... (12)	ALL	0.0692	0.718	1	0.7	1	1
INTEGRIN_COMPLEX (6)	AML	0.073	0.718	1	0.7	1	1
PROTEASOME_COMPLEX (13)	ALL	0.0802	0.718	1	0.7	0.323	1
SMALL_NUCLEAR_RIBONUCLEOPROTEI... (5)	ALL	0.0825	0.718	1	0.719	1	1
AXON (79)	AML	0.0846	0.718	1	0.7	1	1
INTEGRAL_TO_GOLGI_MEMBRANE (50)	ALL	0.0856	0.718	1	0.7	1	1
NEURON_PROJECTION (5)	AML	0.0927	0.718	1	0.812	1	1
CELL_JUNCTION (10)	AML	0.0985	0.718	1	0.7	1	1
VESICULAR_FRACTION (8)	AML	0.101	0.718	1	0.7	1	1
MICROSOME (13)	AML	0.101	0.718	1	0.7	1	1
ER_GOLGI_INTERMEDIATE_COMPARTM... (9)	AML	0.103	0.718	1	0.7	1	1
COLLAGEN (27)	AML	0.105	0.718	1	0.7	1	1
NUCLEAR_CHROMOSOME (48)	ALL	0.106	0.718	1	0.7	1	1
NUCLEAR_SPECK (49)	ALL	0.11	0.718	1	0.7	1	1
SPLICEOSOME (42)	ALL	0.112	0.718	1	0.7	1	1
SPINDLE_MICROTUBULE (89)	ALL	0.114	0.718	1	0.7	1	1
CHROMATIN (8)	ALL	0.118	0.718	1	0.7	1	1
CYTOPLASMIC_MEMBRANE_BOUND_VES... (8)	AML	0.13	0.718	1	0.825	1	1
EXTRACELLULAR_SPACE (77)	AML	0.135	0.718	1	0.7	1	1
INTRINSIC_TO_GOLGI_MEMBRANE (28)	ALL	0.136	0.718	1	0.7	1	1

Table S10: Results for MSigDB c5.cc.v5.0 collection (197 total gene sets after size-based filtering)

Gene set	Direction	GSE p-value	Unweighted q-value	MLRT wFDR	SGSE wFDR	TWT wFDR	MPDT wFDR
STRUCTURAL_MOLECULE_ACTIVITY (23)	AML	0.00435	0.864	0.0369	0.109	0.0381	1
STEROID_HORMONE_RECEPTOR_BINDI... (152)	ALL	0.0164	0.864	0.801	0.515	0.641	0.853
DNA_DIRECTED_DNA_POLYMERASE_AC... (9)	ALL	0.0196	0.864	0.801	0.515	0.641	0.853
PEPTIDE_RECEPTOR_ACTIVITY (21)	AML	0.0206	0.864	0.801	0.515	0.641	0.853
DNA_POLYMERASE_ACTIVITY (171)	ALL	0.0226	0.864	0.956	0.515	0.641	0.954
IONOTROPIC_GLUTAMATE_RECEPTOR_... (17)	AML	0.0317	0.864	0.801	0.515	0.641	0.853
CYTOKINE_ACTIVITY (11)	AML	0.0324	0.864	1	0.687	0.732	0.862
CALCIUM_ION_BINDING (96)	AML	0.0346	0.864	0.959	0.515	0.803	0.853
G_PROTEIN_COUPLED_RECEPTOR_BIN... (17)	AML	0.0362	0.864	0.801	0.515	1	1
OXYGEN_BINDING (36)	AML	0.0365	0.864	0.801	0.606	0.641	0.853
RNA_DEPENDENT_ATPASE_ACTIVITY (7)	ALL	0.0369	0.864	0.801	0.515	0.641	0.853
RNA_HELICASE_ACTIVITY (14)	ALL	0.0385	0.864	0.801	0.515	0.641	0.853
ATP_DEPENDENT_HELICASE_ACTIVIT... (43)	ALL	0.0405	0.864	0.845	0.515	0.641	0.853
ATP_DEPENDENT_DNA_HELICASE_ACT... (40)	ALL	0.042	0.864	0.801	0.606	0.641	0.853
OXIDOREDUCTASE_ACTIVITY_GO_001... (41)	AML	0.0443	0.864	0.959	0.841	0.832	1
CHEMOKINE_ACTIVITY (34)	AML	0.0449	0.864	0.801	0.515	1	0.954
CHEMOKINE_RECEPTOR_BINDING (25)	AML	0.045	0.864	0.801	0.515	1	1
TELOMERIC_DNA_BINDING (143)	ALL	0.0471	0.864	1	0.515	0.937	1
AMINE_RECEPTOR_ACTIVITY (14)	AML	0.0517	0.864	0.959	0.515	0.732	1
RHODOPSIN_LIKE_RECEPTOR_ACTIVI... (18)	AML	0.0529	0.864	0.845	0.606	0.641	1
ATP_DEPENDENT_RNA_HELICASE_ACT... (44)	ALL	0.0543	0.864	0.956	0.606	0.732	0.954
SEROTONIN_RECEPTOR_ACTIVITY (24)	AML	0.0554	0.864	0.845	0.515	0.641	0.853
SPECIFIC_TRANSCRIPTIONAL_REPRE... (38)	ALL	0.063	0.864	1	0.606	0.832	1
CHROMATIN_BINDING (7)	ALL	0.0736	0.864	0.959	0.606	0.654	0.954
HELICASE_ACTIVITY (22)	ALL	0.0761	0.864	0.801	0.606	0.641	0.473

Table S11: Results for MSigDB c5.mf.v5.0 collection (370 total gene sets after size-based filtering)

Gene set	Direction	GSE p-value	Unweighted q-value	MLRT wFDR	SGSE wFDR	TWT wFDR	MPDT wFDR
PIGF_UP.V1_DN (10)	AML	0.0209	0.829	1	0.49	1	1
IL2_UP.V1_UP (14)	AML	0.0352	0.829	0.964	0.49	1	1
BMI1_DN_MEL18_DN.V1_UP (122)	AML	0.0353	0.829	1	0.49	1	1
KRAS.LUNG_UP.V1_UP (103)	AML	0.0414	0.829	0.964	0.49	1	1
CRX_DN.V1_DN (113)	AML	0.0495	0.829	0.964	0.49	1	1
CYCLIN_D1_UP.V1_UP (135)	AML	0.0683	0.829	0.964	0.49	1	1
BCAT_BILD_ET_AL_UP (110)	AML	0.0697	0.829	0.964	0.49	1	1
P53_DN.V2_UP (123)	AML	0.0709	0.829	0.964	0.49	1	1
ALK_DN.V1_UP (98)	AML	0.0767	0.829	1	0.49	1	1
IL15_UP.V1_UP (98)	AML	0.0773	0.829	1	0.49	1	1
BMI1_DN.V1_UP (97)	AML	0.0803	0.829	1	0.534	1	1
ESC_V6.5_UP_EARLY.V1_DN (105)	AML	0.081	0.829	0.964	0.605	1	1
KRAS.LUNG_UP.V1_DN (158)	AML	0.0851	0.829	0.964	0.605	1	1
RPS14_DN.V1_UP (147)	AML	0.0863	0.829	1	0.49	1	1
KRAS.BREAST_UP.V1_UP (17)	AML	0.106	0.829	1	0.49	1	1
KRAS.LUNG.BREAST_UP.V1_UP (9)	AML	0.113	0.829	1	0.605	1	1
MTOR_UP.V1_DN (169)	AML	0.121	0.829	1	0.605	1	1
PRC1_BMI_UP.V1_DN (155)	AML	0.123	0.829	1	0.605	1	1
HINATA_NFKB_IMMU_INF (169)	AML	0.13	0.829	0.964	0.505	1	1
CAHOY_ASTROGLIAL (154)	AML	0.13	0.829	0.964	0.605	1	1
KRAS.LUNG.BREAST_UP.V1_DN (64)	AML	0.133	0.829	1	0.768	1	1
KRAS.KIDNEY_UP.V1_DN (87)	AML	0.134	0.829	0.964	0.605	1	1
NOTCH_DN.V1_DN (90)	AML	0.139	0.829	1	0.605	1	1
KRAS.600.LUNG.BREAST_UP.V1_DN (97)	AML	0.144	0.829	0.964	0.768	1	1
MTOR_UP.N4.V1_DN (100)	ALL	0.147	0.829	0.964	0.697	1	1

Table S12: Results for MSigDB c6.all.v5.0 collection (188 total gene sets after size-based filtering)

Gene set	Direction	GSE p-value	Unweighted q-value	MLRT wFDR	SGSE wFDR	TWT wFDR	MPDT wFDR
GSE10325_BCELL_VS_MYELOID_UP (124)	ALL	0.00225	0.999	0.655	0.711	0.0969	1
GSE29618_BCELL_VS_MONOCYTE_DAY... (130)	ALL	0.00302	0.999	0.655	0.711	0.0969	1
GSE29618_BCELL_VS_MDC_DAY7_FLU... (126)	ALL	0.0046	0.999	0.655	0.729	0.574	1
GSE10325_CD4_TCELL_VS_BCELL_DN (132)	ALL	0.005	0.999	1	0.711	0.574	1
GSE10325_LUPUS_BCELL_VS_LUPUS_... (123)	ALL	0.00563	0.999	0.431	0.711	0.12	1
GSE29618_BCELL_VS_MDC_UP (133)	ALL	0.00719	0.999	0.655	0.955	0.574	1
GSE29618_BCELL_VS_MONOCYTE_UP (108)	ALL	0.00776	0.999	0.655	0.711	0.574	1
GSE29618_BCELL_VS_MONOCYTE_DAY... (143)	AML	0.0137	0.999	0.655	0.711	0.574	1
GSE24634_TREG_VS_TCONV_POST_DA... (123)	AML	0.0162	0.999	1	0.711	1	1
GSE6269_HEALTHY_VS_STREP_AUREU... (133)	AML	0.0168	0.999	0.655	0.711	0.574	1
GSE29618_BCELL_VS_MDC_DAY7_FLU... (126)	AML	0.0171	0.999	0.655	0.711	1	1
GSE6269_HEALTHY_VS_STREP_PNEUM... (134)	AML	0.0174	0.999	0.655	0.711	0.742	1
GSE15767_MED_VS_SCS_MAC_LN_UP (117)	AML	0.0229	0.999	1	0.711	1	1
GSE6269_E_COLI_VS_STREP_AUREUS... (130)	AML	0.0245	0.999	1	0.711	1	1
GSE22886_NAIVE_CD8_TCELL_VS_NE... (122)	AML	0.0295	0.999	1	0.729	1	1
GSE6269_FLU_VS_E_COLI_INF_PBMC... (128)	AML	0.0306	0.999	1	0.711	1	1
GSE29618_MONOCYTE_VS_PDC_UP (126)	AML	0.0333	0.999	0.659	0.711	0.706	1
GSE6269_HEALTHY_VS_STREP_AUREU... (109)	ALL	0.0353	0.999	0.906	0.907	0.592	1
GSE3982_MEMORY_CD4_TCELL_VS_BC... (73)	ALL	0.0361	0.999	1	0.995	1	1
GSE360_CTRL_VS_M_TUBERCULOSIS_... (71)	AML	0.0364	0.999	1	0.711	1	1
GSE11057_EFF_MEM_VS_CENT_MEM_C... (85)	AML	0.0381	0.999	1	0.729	1	1
GSE10325_LUPUS_BCELL_VS_LUPUS_... (88)	AML	0.0384	0.999	0.655	0.711	0.998	1
GSE360_CTRL_VS_L_DONOVANI_DC_D... (75)	AML	0.0403	0.999	1	0.729	1	1
GSE22886_NAIVE_CD4_TCELL_VS_NE... (92)	AML	0.0403	0.999	1	0.758	1	1
GSE3982_CTRL_VS_PMA_STIM_EOSIN... (93)	AML	0.0427	0.999	1	0.711	1	1

Table S13: Results for MSigDB c7.all.v5.0 collection (1910 total gene sets after size-based filtering)

2 Results for v5.0 MSigDB collections and p53 gene expression data

This section contains results for the evaluation detailed in Section 2.6.4 of the main manuscript for all analyzed v5.0 MSigDB collections and the p53 gene expression data set.

Table S14 contains the Spearman rank correlation values between the supervised gene set testing p-values (as computed by the CAMERA method) and the unsupervised gene set testing p-values (as computed by the MLRT, TWT, SGSE or MPDT methods) for each MSigDB collection relative to the p53 mutated phenotype.

Tables S15-S26 below display the 25 gene sets from each MSigDB collection with the most significant p-value from a supervised gene set test relative to the p53 mutated phenotype as computed using the CAMERA method with configuration specified in Section 2.6.5 of the main manuscript. The first column in the table contains the gene set name with the number of genes in the set in parentheses. The second column lists the direction of enrichment, the third column the enrichment significance as computed via CAMERA and the forth column the false discovery rate q-value when all considering all gene sets in the collection as the family of hypotheses. Columns five through eight display the results of a weighted FDR analysis, as detailed in Section 2.6.4 of the main manuscript, using each of the evaluated unsupervised gene set testing methods to compute the weight applied to the CAMERA p-value.

MSigDB Collection	Inter-gene correl.	MLRT	SGSE	TWT	MPDT
c1.all.v5.0	-0.028	0.0472	0.029	0.0743	0.0593
c2.cgp.v5.0	-0.0432	-0.0572	0.163	0.032	0.047
c2.cp.v5.0	-0.0693	-0.0874	0.0446	-0.0404	-0.0657
c3.mir.v5.0	-0.029	0.171	0.365	0.173	0.071
c3.tft.v5.0	0.0412	0.209	0.335	0.185	0.0728
c4.cgn.v5.0	-0.122	-0.00178	0.0135	-0.0587	-0.0171
c4.cm.v5.0	-0.0088	0.0499	0.0127	0.074	-0.0181
c5.bp.v5.0	0.0248	0.0448	0.00684	0.0289	-0.0227
c5.cc.v5.0	-0.114	-0.0954	-0.0788	-0.19	0.0565
c5.mf.v5.0	-0.0187	-0.0983	0.00763	0.0944	-0.12
c6.all.v5.0	0.0476	0.131	0.249	0.186	-0.0298
c7.all.v5.0	-0.0264	0.0656	0.234	0.133	-0.0788

Table S14: Spearman rank correlation values between CAMERA supervised gene set test p-values and either the mean inter-gene correlation or unsupervised gene set test p-values.

Gene set	Direction	GSE p-value	Unweighted q-value	MLRT wFDR	SGSE wFDR	TWT wFDR	MPDT wFDR
chr12q14 (28)	WT	0.00167	0.286	0.302	0.22	0.337	0.469
chr1p33 (28)	MUT	0.00203	0.286	0.418	0.22	0.353	0.469
chrxp11 (51)	MUT	0.00718	0.547	0.252	0.346	0.492	0.469
chr8p23 (5)	WT	0.00899	0.547	0.822	0.456	0.642	0.757
chr16p12 (15)	WT	0.00973	0.547	0.418	0.346	0.492	0.469
chr7p21 (38)	MUT	0.015	0.549	1	0.346	0.803	1
chr12q12 (7)	WT	0.0163	0.549	0.883	0.519	0.967	0.757
chr1p31 (18)	MUT	0.0167	0.549	0.883	0.346	0.642	0.874
chr1p22 (45)	MUT	0.0176	0.549	0.302	0.346	0.337	0.469
chr3q29 (14)	MUT	0.0267	0.635	0.822	0.462	0.642	0.757
chr6p12 (17)	MUT	0.0274	0.635	0.883	0.346	1	1
chr1p32 (5)	MUT	0.0308	0.635	0.883	0.448	0.674	0.757
chr7p11 (10)	MUT	0.0347	0.635	1	0.519	0.967	1
chr1p21 (6)	MUT	0.0352	0.635	1	0.462	1	1
chr3p21 (15)	MUT	0.0374	0.635	1	1	1	1
chr12q13 (5)	WT	0.0386	0.635	0.302	1	0.803	0.85
chr7q22 (13)	WT	0.042	0.635	0.822	0.519	0.337	0.757
chr11q13 (14)	MUT	0.043	0.635	0.302	0.519	0.674	0.757
chr15q23 (39)	WT	0.0466	0.635	1	0.462	1	1
chr1p35 (26)	MUT	0.053	0.635	0.883	0.829	1	1
chr12q24 (50)	WT	0.0539	0.635	0.822	0.53	0.337	0.85
chr11p11 (8)	MUT	0.0539	0.635	1	0.829	1	1
chr2q35 (25)	WT	0.0552	0.635	1	0.853	0.967	1
chr17q22 (7)	WT	0.0579	0.635	1	0.936	0.967	1
chr1q23 (20)	WT	0.0585	0.635	1	0.667	1	1

Table S15: Results for MSigDB c1.all.v5.0 collection (281 total gene sets after size-based filtering)

Gene set	Direction	GSE p-value	Unweighted q-value	MLRT wFDR	SGSE wFDR	TWT wFDR	MPDT wFDR
WARTERS_IR_RESPONSE_5GY (17)	WT	6.82e-09	2.02e-05	1.33e-05	4.75e-05	1.2e-05	0.000201
AMUNDSON_DNA_DAMAGE_RESPONSE_T... (28)	WT	7.35e-06	0.00982	0.0119	0.0311	0.0357	0.251
WARTERS_RESPONSE_TO_IR_SKIN (14)	WT	9.94e-06	0.00982	0.00542	0.0492	0.00872	0.338
NUNODA_RESPONSE_TO_DASATINIB_I... (18)	WT	1.92e-05	0.0142	0.015	0.0311	0.0481	0.239
DITTMER_PTHLH_TARGETS_UP (53)	MUT	0.000115	0.0591	1	0.0331	0.0481	0.0277
KUMAMOTO_RESPONSE_TO_NUTLIN_3A... (31)	WT	0.00012	0.0591	0.446	0.125	0.565	1
IVANOV_MUTATED_IN_COLON_CANCER (35)	MUT	0.000205	0.0867	0.464	0.052	0.567	0.584
WELCSH_BRCA1_TARGETS_UP (37)	MUT	0.000448	0.166	1	0.052	0.131	0.0717
GENTILE_UV_LOW_DOSE_UP (6)	WT	0.000718	0.237	0.575	0.271	0.628	1
TAYLOR_METHYLATED_IN_ACUTE_LYM... (151)	MUT	0.000804	0.238	0.166	0.168	0.201	0.831
APRELIKOVA_BRCA1_TARGETS (131)	MUT	0.000914	0.247	0.166	0.143	0.201	0.693
KAAB_FAILED_HEART_ATRIUM_DN (71)	MUT	0.00108	0.266	1	0.096	0.21	0.129
SCHAVOLT_TARGETS_OF_TP53_AND_T... (16)	WT	0.00122	0.278	0.464	0.421	0.628	0.774
CAMPS_COLON_CANCER_COPY_NUMBER... (6)	MUT	0.00133	0.281	0.589	0.374	0.628	1
KIM_MYCL1_AMPLIFICATION_TARGET... (17)	MUT	0.00183	0.363	0.753	0.271	0.765	1
BROWNE_HCMV_INFECTION_2HR_DN (9)	MUT	0.00226	0.395	0.315	0.399	0.368	0.829
CEBALLOS_TARGETS_OF_TP53_AND_M... (11)	WT	0.00231	0.395	0.315	0.677	0.368	0.86
HOWLIN_CITED1_TARGETS_2_DN (18)	MUT	0.0024	0.395	0.735	0.374	0.918	1
LANDIS_ERBB2_BREAST_TUMORS_324... (89)	MUT	0.00281	0.439	1	0.262	0.411	0.338
INGA_TP53_TARGETS (82)	WT	0.00302	0.448	0.61	0.463	0.765	0.938
RAMPON_ENRICHED_LEARNING_ENVIR... (135)	MUT	0.00354	0.499	0.735	0.336	0.792	1
GEISS_RESPONSE_TO_DSRNA_DN (162)	MUT	0.00377	0.509	0.753	0.336	0.789	1
GROSS_HYPOXIA_VIA_HIF1A_DN (49)	MUT	0.00435	0.524	1	0.421	0.526	0.646
LEE_AGING_MUSCLE_DN (82)	MUT	0.00446	0.524	0.446	0.336	0.526	1
BARIS_THYROID_CANCER_DN (197)	MUT	0.0045	0.524	0.446	0.336	0.526	0.951

Table S16: Results for MSigDB c2.cgp.v5.0 collection (2966 total gene sets after size-based filtering)

Gene set	Direction	GSE p-value	Unweighted q-value	MLRT wFDR	SGSE wFDR	TWT wFDR	MPDT wFDR
PID_NCADHERIN_PATHWAY (49)	MUT	0.000185	0.241	0.848	0.174	0.813	0.752
REACTOME_RAF_MAP_KINASE_CASCAD... (27)	MUT	0.000572	0.264	0.848	0.482	0.813	0.752
PID_EPHB_FWD_PATHWAY (20)	MUT	0.000657	0.264	0.848	0.359	0.813	0.752
BIOCARTA_P53_PATHWAY (12)	WT	0.000813	0.264	0.848	0.549	0.813	0.752
BIOCARTA_FMLP_PATHWAY (29)	MUT	0.00114	0.298	0.848	0.303	0.859	0.752
BIOCARTA_RAS_PATHWAY (22)	MUT	0.00215	0.466	0.848	0.482	1	0.752
REACTOME_ARMS_MEDIATED_ACTIVAT... (12)	MUT	0.00299	0.555	0.848	0.482	1	0.752
REACTOME_SEMA3A_PAK_DEPENDENT_... (34)	MUT	0.00346	0.558	1	0.482	0.813	0.874
PID_WNT_NONCANONICAL_PATHWAY (14)	MUT	0.00427	0.558	0.897	0.482	1	0.752
BIOCARTA_HSP27_PATHWAY (11)	WT	0.00432	0.558	0.848	0.549	0.813	0.752
PID_AVB3_OPN_PATHWAY (35)	MUT	0.00621	0.558	1	0.482	0.813	1
BIOCARTA_TALL1_PATHWAY (80)	MUT	0.00708	0.558	0.93	0.549	0.827	0.858
REACTOME_SHC1_EVENTS_IN_EGFR_S... (112)	MUT	0.00731	0.558	1	0.549	1	1
PID_ECADHERIN_KERATINOCYTE_PAT... (62)	MUT	0.00734	0.558	0.848	0.482	0.813	0.752
REACTOME_ASPARAGINE_N_LINKED_G... (25)	MUT	0.00737	0.558	1	0.549	1	1
BIOCARTA_ASBCELL_PATHWAY (25)	WT	0.0074	0.558	0.848	0.613	0.813	0.874
REACTOME_PROLONGED_ERK_ACTIVAT... (22)	MUT	0.00757	0.558	0.94	0.613	1	0.752
BIOCARTA_BCR_PATHWAY (36)	MUT	0.00943	0.558	0.848	0.549	1	0.813
ST_PHOSPHOINOSITIDE_3_KINASE_P... (7)	MUT	0.00988	0.558	1	0.559	1	1
BIOCARTA_IL4_PATHWAY (21)	WT	0.0101	0.558	0.848	0.554	1	1
BIOCARTA_GRANULOCYTES_PATHWAY (40)	WT	0.0108	0.558	0.848	0.694	0.813	0.752
REACTOME_PEROXISOMAL_LIPID_MET... (19)	MUT	0.0114	0.558	1	0.549	0.95	1
BIOCARTA_IGF1_PATHWAY (34)	MUT	0.0117	0.558	1	0.613	1	0.874
SA_B_CELL_RECEPTOR_COMPLEXES (17)	MUT	0.0124	0.558	1	0.583	1	1
REACTOME_GLUCURONIDATION (28)	WT	0.0125	0.558	0.848	0.792	0.813	1

Table S17: Results for MSigDB c2.cp.v5.0 collection (1300 total gene sets after size-based filtering)

Gene set	Direction	GSE p-value	Unweighted q-value	MLRT wFDR	SGSE wFDR	TWT wFDR	MPDT wFDR
GTGCCAT,MIR-183 (161)	MUT	0.016	0.618	0.565	0.444	0.642	0.791
ATTCTTT,MIR-186 (180)	MUT	0.0192	0.618	0.565	0.444	0.642	0.774
AAAGGAT,MIR-501 (141)	MUT	0.0245	0.618	0.565	0.444	0.642	1
ATAAGCT,MIR-21 (10)	MUT	0.0267	0.618	0.565	0.444	0.642	1
CCTGTGA,MIR-513 (146)	MUT	0.0303	0.618	0.565	0.444	0.642	1
AGGAGTG,MIR-483 (141)	MUT	0.0307	0.618	0.565	0.444	0.642	1
CCTGAGT,MIR-510 (13)	MUT	0.0331	0.618	0.565	0.444	0.642	1
TCCGTCC,MIR-184 (25)	MUT	0.0349	0.618	0.565	0.444	0.642	1
GAGCCTG,MIR-484 (200)	MUT	0.041	0.618	0.565	0.444	0.642	1
ACACTAC,MIR-142-3P (8)	MUT	0.0534	0.618	0.565	0.444	0.642	1
GGATCCG,MIR-127 (159)	MUT	0.0538	0.618	0.661	0.444	1	1
TTTTGAG,MIR-373 (34)	MUT	0.0546	0.618	0.565	0.444	0.642	1
GCAAAAA,MIR-129 (115)	MUT	0.065	0.618	0.565	0.488	0.748	1
CAGCTTT,MIR-320 (173)	MUT	0.0673	0.618	0.565	0.444	0.642	1
TTGCCAA,MIR-182 (100)	MUT	0.0674	0.618	0.565	0.444	0.642	1
GCGCCTT,MIR-525,MIR-524 (156)	MUT	0.0688	0.618	0.578	0.741	0.642	1
ACATTCC,MIR-1,MIR-206 (155)	MUT	0.069	0.618	0.565	0.444	0.642	1
TCTGATA,MIR-361 (40)	MUT	0.0741	0.618	0.565	0.444	1	1
CCACACA,MIR-147 (33)	MUT	0.0835	0.618	0.661	0.444	0.959	1
AGTCTAG,MIR-151 (180)	MUT	0.0855	0.618	1	0.588	1	1
ATAACCT,MIR-154 (28)	MUT	0.086	0.618	0.578	0.444	0.642	1
GTGACTT,MIR-224 (27)	MUT	0.0869	0.618	0.565	0.444	0.642	1
ACACTCC,MIR-122A (62)	MUT	0.0896	0.618	0.565	0.588	0.642	1
TGTATGA,MIR-485-3P (7)	MUT	0.0914	0.618	0.565	0.444	0.642	1
GTGTGAG,MIR-342 (69)	MUT	0.0954	0.618	0.565	0.838	0.959	1

Table S18: Results for MSigDB c3.mir.v5.0 collection (201 total gene sets after size-based filtering)

Gene set	Direction	GSE p-value	Unweighted q-value	MLRT wFDR	SGSE wFDR	TWT wFDR	MPDT wFDR
CCAATNNSNNNGCG_UNKNOWN (114)	MUT	0.00344	0.852	0.727	0.566	1	0.618
V$USF2_Q6 (200)	MUT	0.00586	0.852	0.727	0.566	0.8	0.533
V$ETF_Q6 (121)	MUT	0.00763	0.852	0.727	0.566	0.871	0.973
V$AP1_Q6_01 (133)	MUT	0.0138	0.852	0.727	0.566	1	0.973
GGCKCATGS_UNKNOWN (127)	MUT	0.0143	0.852	1	0.659	0.948	1
V$PAX8_01 (130)	MUT	0.0241	0.852	0.901	0.566	0.899	0.998
V$TCF11MAFG_01 (76)	MUT	0.0246	0.852	1	0.566	1	1
V$HIF1_Q5 (128)	MUT	0.0276	0.852	0.901	0.659	1	0.998
V$MAZR_01 (124)	MUT	0.0279	0.852	1	0.66	1	1
WWTAAGGC_UNKNOWN (15)	MUT	0.0295	0.852	1	0.566	1	0.998
MCAATNNNNNGCG_UNKNOWN (148)	MUT	0.03	0.852	1	0.659	0.948	1
V$ALPHACP1_01 (22)	MUT	0.0317	0.852	0.858	0.566	0.944	0.998
V$MYC_Q2 (44)	MUT	0.0326	0.852	0.878	0.627	1	0.491
V$MIF1_01 (114)	MUT	0.0337	0.852	1	0.566	1	1
ACAWNRNSRCGG_UNKNOWN (132)	MUT	0.0357	0.852	0.958	0.566	0.813	1
V$CREB_Q4 (124)	MUT	0.0362	0.852	1	0.566	1	0.998
V$AP1_C (62)	MUT	0.0392	0.852	0.951	0.566	1	1
V$SP1_01 (132)	MUT	0.0401	0.852	1	0.645	1	0.998
V$ATF4_Q2 (151)	MUT	0.0417	0.852	1	0.659	1	1
V$AP1_01 (130)	MUT	0.042	0.852	1	0.566	1	1
V$AML_Q6 (84)	WT	0.0448	0.852	1	0.723	1	1
V$NF1_Q6 (133)	MUT	0.0455	0.852	1	0.659	1	1
V$CREB_Q2_01 (137)	MUT	0.046	0.852	1	0.566	1	0.491
V$NF1_Q6_01 (115)	MUT	0.0474	0.852	1	0.862	1	0.491
V$BACH1_01 (119)	MUT	0.0495	0.852	1	0.566	1	1

Table S19: Results for MSigDB c3.tft.v5.0 collection (565 total gene sets after size-based filtering)

Gene set	Direction	GSE p-value	Unweighted q-value	MLRT wFDR	SGSE wFDR	TWT wFDR	MPDT wFDR
MORF_PAPSS1 (156)	MUT	0.0199	0.708	0.735	0.781	0.725	0.794
GCM_DEAF1 (68)	MUT	0.02	0.708	1	0.781	0.989	1
GCM_RAB10 (178)	MUT	0.0504	0.708	1	0.781	1	1
GCM_PTPRU (154)	WT	0.0535	0.708	1	0.837	1	1
MORF_PRKAG1 (40)	MUT	0.0576	0.708	0.735	0.781	0.821	1
MORF_RAGE (158)	WT	0.0577	0.708	0.735	0.781	0.725	1
MORF_DAP (78)	MUT	0.0583	0.708	0.883	0.781	1	0.986
MORF_JAK3 (46)	WT	0.0617	0.708	0.735	0.781	0.725	1
GNF2_SERPINB5 (135)	MUT	0.0658	0.708	1	0.781	0.982	1
MORF_LMO1 (53)	WT	0.0678	0.708	1	0.781	0.725	1
MORF_CASP2 (70)	WT	0.0679	0.708	0.735	0.781	0.725	1
GCM_CRKL (96)	MUT	0.068	0.708	1	0.781	1	1
GCM_BAG5 (77)	MUT	0.0778	0.708	1	0.781	1	1
GNF2_CD14 (56)	WT	0.0792	0.708	1	0.781	1	1
GCM_ZNF198 (184)	MUT	0.0828	0.708	1	0.781	0.997	1
MORF_DDX11 (56)	WT	0.0868	0.708	0.735	0.781	0.725	1
GCM_NCAM1 (48)	MUT	0.0872	0.708	1	0.781	1	1
GCM_ATM (142)	WT	0.0886	0.708	1	0.781	1	1
MORF_PML (195)	WT	0.0987	0.708	0.735	0.781	0.725	1
GCM_MAP1B (49)	MUT	0.102	0.708	1	0.781	1	1
GCM_HMGA2 (192)	WT	0.103	0.708	1	0.837	1	1
MORF_FDXR (135)	WT	0.103	0.708	0.735	0.781	0.725	1
GCM_MYCL1 (74)	WT	0.105	0.708	1	0.781	1	1
MORF_LTK (68)	WT	0.108	0.708	0.735	0.781	0.725	1
MORF_HEAB (148)	WT	0.111	0.708	1	0.818	1	1

Table S20: Results for MSigDB c4.cgn.v5.0 collection (401 total gene sets after size-based filtering)

Gene set	Direction	GSE p-value	Unweighted q-value	MLRT wFDR	SGSE wFDR	TWT wFDR	MPDT wFDR
MODULE_503 (17)	MUT	0.00263	0.756	0.638	0.309	0.799	0.723
MODULE_576 (188)	MUT	0.00522	0.756	0.767	0.457	0.854	1
MODULE_105 (71)	MUT	0.00678	0.756	0.612	0.309	0.638	0.723
MODULE_488 (15)	WT	0.00982	0.756	0.767	0.457	1	0.855
MODULE_263 (72)	WT	0.0101	0.756	0.612	0.967	1	0.822
MODULE_86 (10)	MUT	0.018	0.858	1	0.482	1	0.822
MODULE_421 (38)	MUT	0.029	0.858	0.612	0.967	0.638	0.659
MODULE_326 (13)	WT	0.0306	0.858	1	0.967	1	1
MODULE_87 (63)	MUT	0.033	0.858	0.892	0.967	1	1
MODULE_147 (31)	MUT	0.0436	0.858	1	0.967	1	1
MODULE_416 (26)	MUT	0.0472	0.858	1	0.967	1	1
MODULE_108 (182)	WT	0.0478	0.858	1	0.967	1	1
MODULE_289 (13)	WT	0.0632	0.858	1	0.967	1	1
MODULE_462 (115)	WT	0.0692	0.858	1	0.967	1	1
MODULE_356 (8)	MUT	0.0695	0.858	1	0.967	1	1
MODULE_195 (78)	MUT	0.0708	0.858	1	0.967	1	1
MODULE_402 (21)	WT	0.0721	0.858	1	0.967	0.638	1
MODULE_243 (92)	MUT	0.0829	0.858	1	0.967	1	1
MODULE_438 (167)	MUT	0.0851	0.858	0.612	0.967	1	0.987
MODULE_222 (150)	MUT	0.0876	0.858	0.612	0.967	0.638	0.659
MODULE_397 (105)	MUT	0.091	0.858	1	0.986	0.638	1
MODULE_188 (13)	WT	0.0914	0.858	1	1	1	1
MODULE_297 (35)	MUT	0.0932	0.858	0.612	0.967	1	1
MODULE_322 (173)	MUT	0.0936	0.858	1	1	1	1
MODULE_334 (12)	MUT	0.0954	0.858	1	1	1	1

Table S21: Results for MSigDB c4.cm.v5.0 collection (375 total gene sets after size-based filtering)

Gene set	Direction	GSE p-value	Unweighted q-value	MLRT wFDR	SGSE wFDR	TWT wFDR	MPDT wFDR
RESPONSE_TO_TOXIN (5)	WT	0.00316	0.853	0.733	0.436	0.813	0.827
PEROXISOME_ORGANIZATION_AND_BI... (175)	MUT	0.00331	0.853	0.733	0.436	0.813	0.715
REGULATION_OF_RAS_PROTEIN_SIGN... (9)	MUT	0.00384	0.853	1	0.436	1	0.715
INDUCTION_OF_APOPTOSIS_BY_INTR... (85)	WT	0.00725	0.853	0.75	0.436	0.813	1
ONE_CARBON_COMPOUND_METABOLIC_... (10)	MUT	0.00803	0.853	0.733	0.436	0.813	0.715
NEGATIVE_REGULATION_OF_DNA_BIN... (7)	MUT	0.00975	0.853	0.733	0.436	0.813	0.715
NEGATIVE_REGULATION_OF_BINDING (25)	MUT	0.00975	0.853	0.733	0.436	0.813	0.715
NEGATIVE_REGULATION_OF_TRANSCR... (10)	MUT	0.0106	0.853	0.733	0.436	0.813	0.715
PROTEIN_TRANSPORT (94)	MUT	0.0113	0.853	0.733	0.436	0.915	0.715
REGULATION_OF_SMALL_GTPASE_MED... (21)	MUT	0.0115	0.853	1	0.436	1	1
INTRACELLULAR_TRANSPORT (35)	MUT	0.0137	0.923	0.733	0.436	0.878	0.715
NEGATIVE_REGULATION_OF_MYELOID... (71)	WT	0.0179	0.933	1	1	1	1
INTRACELLULAR_PROTEIN_TRANSPOR... (18)	MUT	0.0191	0.933	0.733	0.436	1	0.715
ESTABLISHMENT_OF_PROTEIN_LOCAL... (15)	MUT	0.0196	0.933	0.733	0.436	1	0.715
ENDOTHELIAL_CELL_MIGRATION (12)	MUT	0.0197	0.933	0.792	0.676	1	1
REGULATION_OF_MYELOID_CELL_DIF... (42)	WT	0.0204	0.933	1	1	1	1
SYNAPTOGENESIS (5)	WT	0.0261	0.933	1	1	1	1
MACROMOLECULE_LOCALIZATION (18)	MUT	0.0261	0.933	0.733	0.436	0.845	0.715
DEFENSE_RESPONSE_TO_VIRUS (23)	WT	0.0262	0.933	1	1	1	1
REGULATION_OF_DEFENSE_RESPONSE (22)	WT	0.0283	0.933	1	1	1	1
REGULATION_OF_MITOTIC_CELL_CYC... (126)	MUT	0.0287	0.933	1	1	1	1
PHOTOTRANSDUCTION (10)	WT	0.0366	0.933	1	1	1	1
SPERM_MOTILITY (8)	WT	0.0406	0.933	1	1	1	1
PROTEIN_TARGETING (19)	MUT	0.0418	0.933	1	0.814	1	0.859
REGULATION_OF_KINASE_ACTIVITY (38)	MUT	0.043	0.933	0.887	1	1	1

Table S22: Results for MSigDB c5.bp.v5.0 collection (743 total gene sets after size-based filtering)

Gene set	Direction	GSE p-value	Unweighted q-value	MLRT wFDR	SGSE wFDR	TWT wFDR	MPDT wFDR
HISTONE_DEACETYLASE_COMPLEX (167)	MUT	0.00393	0.486	0.933	0.432	0.882	0.552
INSOLUBLE_FRACTION (6)	WT	0.00662	0.486	1	0.461	1	1
ENDOMEMBRANE_SYSTEM (11)	MUT	0.0106	0.486	0.933	0.432	0.882	0.576
MICROBODY (16)	MUT	0.0123	0.486	1	0.432	1	1
PEROXISOME (27)	MUT	0.0123	0.486	1	0.432	1	1
GOLGI_MEMBRANE (12)	MUT	0.0151	0.497	1	0.605	1	1
PEROXISOMAL_MEMBRANE (6)	MUT	0.0221	0.533	1	0.461	1	1
MICROBODY_MEMBRANE (13)	MUT	0.0221	0.533	1	0.461	1	1
CELL_CORTEX (5)	MUT	0.0244	0.533	0.933	0.461	1	1
PEROXISOMAL_PART (80)	MUT	0.037	0.639	1	0.461	1	1
MICROBODY_PART (50)	MUT	0.037	0.639	1	0.461	1	1
TRANSCRIPTION_FACTOR_COMPLEX (5)	MUT	0.0389	0.639	1	0.461	0.882	1
INTRINSIC_TO_GOLGI_MEMBRANE (10)	MUT	0.0488	0.74	1	0.461	1	1
GOLGI_APPARATUS_PART (8)	MUT	0.0568	0.747	1	0.75	1	1
MICROVILLUS (13)	MUT	0.0574	0.747	1	0.734	1	1
ORGANELLE_MEMBRANE (9)	MUT	0.0607	0.747	0.933	0.461	1	0.552
ENDOPLASMIC_RETICULUM (27)	MUT	0.0697	0.807	1	0.605	1	1
GOLGI_APPARATUS (48)	MUT	0.0738	0.807	1	0.605	1	1
NUCLEAR_ENVELOPE_ENDOPLASMIC_R... (49)	MUT	0.0952	0.901	1	0.996	1	1
VESICLE_MEMBRANE (42)	MUT	0.103	0.901	1	0.793	1	1
CELL_CORTEX_PART (89)	MUT	0.11	0.901	1	0.996	1	1
INTEGRAL_TO_GOLGI_MEMBRANE (8)	MUT	0.115	0.901	1	0.793	1	1
ENDOPLASMIC_RETICULUM_PART (8)	MUT	0.118	0.901	1	0.845	1	1
INTEGRAL_TO_ORGANELLE_MEMBRANE (77)	MUT	0.131	0.901	1	1	1	1
LATE_ENDOSOME (29)	MUT	0.134	0.901	1	0.793	1	1

Table S23: Results for MSigDB c5.cc.v5.0 collection (197 total gene sets after size-based filtering)

Gene set	Direction	GSE p-value	Unweighted q-value	MLRT wFDR	SGSE wFDR	TWT wFDR	MPDT wFDR
PROTEIN_DEACETYLASE_ACTIVITY (23)	MUT	0.00296	0.949	0.559	0.752	0.832	0.723
DEACETYLASE_ACTIVITY (152)	MUT	0.0137	0.949	0.908	1	0.953	0.723
CHEMOKINE_RECEPTOR_BINDING (10)	WT	0.0145	0.949	0.938	1	1	1
G_PROTEIN_COUPLED_RECEPTOR_BIN... (21)	WT	0.0172	0.949	1	1	1	1
CHEMOKINE_ACTIVITY (171)	WT	0.0192	0.949	0.938	1	1	1
SIGNAL_SEQUENCE_BINDING (17)	MUT	0.0202	0.949	1	1	1	1
ACTIN_FILAMENT_BINDING (11)	MUT	0.0205	0.949	0.908	1	0.953	0.723
INORGANIC_ANION_TRANSMEMBRANE_... (96)	WT	0.023	0.949	1	1	1	1
CYCLIC_NUCLEOTIDE_PHOSPHODIEST... (17)	WT	0.0373	0.949	1	1	1	1
3_5_CYCLIC_NUCLEOTIDE_PHOSPHOD... (36)	WT	0.038	0.949	1	1	1	1
PHOSPHATE_TRANSMEMBRANE_TRANSP... (7)	WT	0.0415	0.949	1	1	1	1
CYTOKINE_ACTIVITY (14)	WT	0.0425	0.949	1	1	1	1
STEROL_BINDING (43)	MUT	0.0459	0.949	1	1	1	1
ACETYLGLUCOSAMINYLTRANSFERASE_... (41)	MUT	0.0513	0.949	1	1	1	1
ACTIN_BINDING (41)	MUT	0.0597	0.949	1	1	1	1
CASPASE_REGULATOR_ACTIVITY (35)	MUT	0.0635	0.949	1	1	1	1
TRANSFERASE_ACTIVITY_TRANSFERR... (25)	MUT	0.0693	0.949	1	1	1	1
PHOSPHATASE_REGULATOR_ACTIVITY (144)	MUT	0.0699	0.949	1	1	1	1
AMINE_RECEPTOR_ACTIVITY (14)	WT	0.0784	0.949	1	1	1	1
RHO_GUANYL_NUCLEOTIDE_EXCHANGE... (18)	WT	0.0825	0.949	1	1	1	1
PROTEIN_TRANSPORTER_ACTIVITY (46)	WT	0.0888	0.949	1	1	1	1
CYTOSKELETAL_PROTEIN_BINDING (24)	MUT	0.0889	0.949	0.559	1	1	1
NUCLEOSIDE_TRIPHOSPHATASE_ACTI... (38)	MUT	0.0897	0.949	1	1	1	1
PHOSPHORUS_OXYGEN_LYASE_ACTIVI... (7)	WT	0.0904	0.949	1	1	1	1
HYDROLASE_ACTIVITY_ACTING_ON_A... (22)	MUT	0.092	0.949	1	1	0.832	1

Table S24: Results for MSigDB c5.mf.v5.0 collection (370 total gene sets after size-based filtering)

Gene set	Direction	GSE p-value	Unweighted q-value	MLRT wFDR	SGSE wFDR	TWT wFDR	MPDT wFDR
P53_DN.V1_UP (10)	MUT	5.21e-09	9.79e-07	1.43e-07	6.53e-07	1.31e-06	1.09e-05
P53_DN.V1_DN (14)	WT	8.67e-07	8.15e-05	1.19e-05	9.56e-05	6.96e-06	1.09e-05
RB_P130_DN.V1_DN (123)	MUT	0.0342	0.914	1	0.505	1	1
BCAT.100_UP.V1_UP (103)	MUT	0.0385	0.914	1	0.517	1	1
VEGF_A_UP.V1_DN (114)	MUT	0.0432	0.914	0.315	0.505	0.231	1
EGFR_UP.V1_DN (136)	MUT	0.046	0.914	0.315	0.505	1	0.989
RB_DN.V1_DN (110)	MUT	0.056	0.914	1	0.505	1	1
CORDENONSI_YAP_CONSERVED_SIGNA... (124)	MUT	0.0593	0.914	0.324	0.505	0.238	0.208
RAF_UP.V1_UP (98)	MUT	0.0597	0.914	0.873	0.505	1	1
SRC_UP.V1_UP (98)	WT	0.0604	0.914	1	1	1	1
RPS14_DN.V1_DN (98)	MUT	0.0651	0.914	1	0.734	1	0.208
HOXA9_DN.V1_DN (105)	MUT	0.0685	0.914	1	0.505	1	1
CSR_EARLY_UP.V1_UP (159)	MUT	0.0719	0.914	0.958	0.505	1	1
TBK1.DF_DN (150)	MUT	0.0797	0.914	1	0.56	1	1
EGFR_UP.V1_UP (17)	MUT	0.0832	0.914	0.332	0.517	1	1
MEK_UP.V1_UP (9)	MUT	0.085	0.914	0.332	0.505	1	1
GCNP_SHH_UP_EARLY.V1_UP (170)	MUT	0.0875	0.914	0.958	1	0.281	1
ESC_J1_UP_EARLY.V1_DN (156)	MUT	0.0899	0.914	1	0.734	1	1
ERB2_UP.V1_UP (170)	MUT	0.103	0.914	1	0.56	1	1
KRAS.300_UP.V1_UP (155)	WT	0.108	0.914	1	0.994	1	1
BRCA1_DN.V1_UP (64)	WT	0.125	0.914	1	1	1	1
AKT_UP.V1_UP (87)	MUT	0.127	0.914	1	0.821	1	1
ERB2_UP.V1_DN (90)	MUT	0.13	0.914	0.444	0.56	1	1
STK33_SKM_DN (97)	MUT	0.139	0.914	1	0.56	1	1
ALK_DN.V1_UP (100)	WT	0.146	0.914	1	1	1	1

Table S25: Results for MSigDB c6.all.v5.0 collection (188 total gene sets after size-based filtering)

Gene set	Direction	GSE p-value	Unweighted q-value	MLRT wFDR	SGSE wFDR	TWT wFDR	MPDT wFDR
GSE3982_CTRL_VS_LPS_48H_DC_UP (125)	MUT	0.000681	0.727	0.99	0.481	0.947	1
GSE17721_LPS_VS_CPG_4H_BMDM_DN (131)	MUT	0.00112	0.727	0.99	0.481	0.897	1
GSE17721_LPS_VS_GARDIQUIMOD_12... (127)	MUT	0.00114	0.727	0.99	0.481	0.9	1
GSE1432_CTRL_VS_IFNG_24H_MICRO... (132)	MUT	0.00274	0.995	0.99	0.733	0.897	1
GSE15324_ELF4_KO_VS_WT_ACTIVAT... (124)	MUT	0.00401	0.995	0.99	0.575	0.9	1
GSE3982_MAC_VS_NEUTROPHIL_UP (134)	MUT	0.00422	0.995	0.99	0.481	0.897	1
GSE29617_CTRL_VS_DAY7_TIV_FLU_... (108)	WT	0.0055	0.995	0.99	0.797	1	1
GSE37416_12H_VS_48H_F_TULARENS... (144)	MUT	0.00577	0.995	0.99	0.582	0.9	1
GSE26928_NAIVE_VS_CXCR5_POS_CD... (123)	MUT	0.00765	0.995	0.99	0.673	0.947	1
GSE37416_CTRL_VS_6H_F_TULARENS... (134)	MUT	0.00801	0.995	0.99	0.673	0.897	1
GSE11864_UNTREATED_VS_CSF1_PAM... (126)	MUT	0.00963	0.995	0.99	0.733	0.9	1
GSE17721_PAM3CSK4_VS_CPG_6H_BM... (135)	MUT	0.00969	0.995	0.99	0.733	0.897	1
GSE32423_IL7_VS_IL7_IL4_MEMORY... (117)	MUT	0.00974	0.995	0.99	0.733	1	1
GSE360_CTRL_VS_L_DONOVANI_MAC_... (130)	MUT	0.0101	0.995	0.99	0.733	0.897	1
GSE2706_UNSTIM_VS_8H_LPS_AND_R... (122)	MUT	0.0109	0.995	0.99	0.733	0.9	1
GSE37416_CTRL_VS_12H_F_TULAREN... (128)	MUT	0.0115	0.995	0.99	0.733	1	1
GSE10463_CD40L_AND_VA347_VS_CD... (126)	MUT	0.0122	0.995	0.99	0.733	0.981	1
GSE22886_NAIVE_BCELL_VS_BLOOD_... (110)	MUT	0.0144	0.995	0.99	0.673	0.9	1
GSE18791_UNSTIM_VS_NEWCATSLE_V... (74)	MUT	0.0148	0.995	0.99	0.733	1	1
GSE14308_TH2_VS_NAIVE_CD4_TCEL... (71)	MUT	0.0151	0.995	0.99	0.733	0.897	1
GSE17721_POLYIC_VS_GARDIQUIMOD... (85)	MUT	0.0155	0.995	0.99	0.673	0.897	1
GSE17721_LPS_VS_POLYIC_4H_BMDM... (88)	MUT	0.0164	0.995	1	0.733	1	1
GSE20366_EX_VIVO_VS_HOMEOSTATI... (76)	MUT	0.0166	0.995	1	0.874	1	1
GSE17721_LPS_VS_PAM3CSK4_4H_BM... (92)	MUT	0.0204	0.995	0.99	0.733	0.947	1
GSE15930_STIM_VS_STIM_AND_TRIC... (95)	MUT	0.0205	0.995	0.99	0.733	0.908	1

Table S26: Results for MSigDB c7.all.v5.0 collection (1910 total gene sets after size-based filtering)
